# Notch Is Required in Adult *Drosophila* Sensory Neurons for Morphological and Functional Plasticity of the Olfactory Circuit

**DOI:** 10.1371/journal.pgen.1005244

**Published:** 2015-05-26

**Authors:** Simon Kidd, Gary Struhl, Toby Lieber

**Affiliations:** Department of Genetics and Development, Columbia University College of Physicians and Surgeons, New York, New York, United States of America; New York University, UNITED STATES

## Abstract

Olfactory receptor neurons (ORNs) convey odor information to the central brain, but like other sensory neurons were thought to play a passive role in memory formation and storage. Here we show that Notch, part of an evolutionarily conserved intercellular signaling pathway, is required in adult *Drosophila* ORNs for the structural and functional plasticity of olfactory glomeruli that is induced by chronic odor exposure. Specifically, we show that Notch activity in ORNs is necessary for the odor specific increase in the volume of glomeruli that occurs as a consequence of prolonged odor exposure. Calcium imaging experiments indicate that Notch in ORNs is also required for the chronic odor induced changes in the physiology of ORNs and the ensuing changes in the physiological response of their second order projection neurons (PNs). We further show that Notch in ORNs acts by both canonical cleavage-dependent and non-canonical cleavage-independent pathways. The Notch ligand Delta (Dl) in PNs switches the balance between the pathways. These data define a circuit whereby, in conjunction with odor, N activity in the periphery regulates the activity of neurons in the central brain and Dl in the central brain regulates N activity in the periphery. Our work highlights the importance of experience dependent plasticity at the first olfactory synapse.

## Author Summary

Appropriate behavioral responses to changing environmental signals, such as odors, are essential for an organism’s survival. In *Drosophila* odors are detected by olfactory receptor neurons (ORNs) that synapse with second order projection neurons (PNs) and local interneurons in morphologically identifiable neuropils in the antennal lobe called glomeruli. Chronic odor exposure leads to changes in animal behavior as well as to changes in the activity of neurons in the olfactory circuit and increases in the volume of glomeruli. Here, we establish that Notch, an evolutionarily conserved transmembrane receptor that plays profound and pervasive roles in animal development, is required in adult *Drosophila* ORNs for functional and morphological plasticity in response to chronic odor exposure. These findings are significant because they point to a role for Notch in regulating activity dependent plasticity. Furthermore, we show that in regulating the odor dependent change in glomerular volume, Notch acts by both non-canonical, cleavage-independent and canonical, cleavage-dependent mechanisms, with the Notch ligand Delta in PNs switching the balance between the pathways. Because both the Notch pathway and the processing of olfactory information are highly conserved between flies and vertebrates these findings are likely to be of general relevance.

## Introduction

Appropriate behavioral responses to changing environments are essential for an organism’s survival. Odors are key environmental signals. In response to chronic odor exposure, depending on the valence of the conditioning odor and the context of odor delivery, an animal will either exhibit decreased responsiveness or enhanced attraction to subsequent presentations of the same odor [1–6].

**Fig 1:**
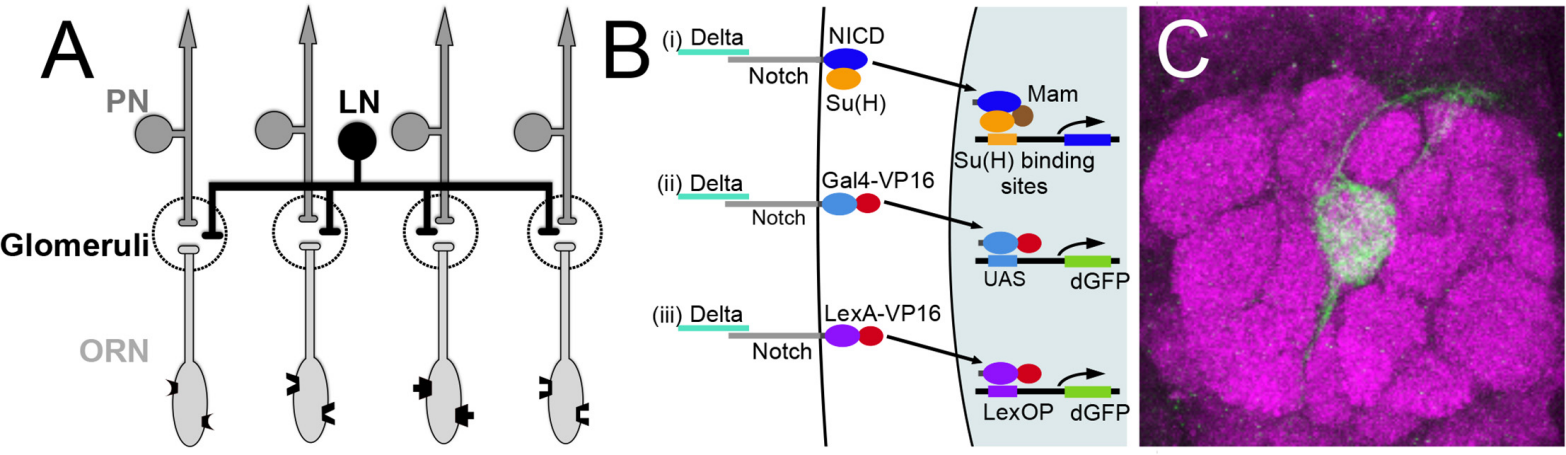
Olfactory circuit and N reporter assay. (A) Diagram of the olfactory circuit. ORN, olfactory receptor neuron; PN, projection neuron; LN, local interneuron. (B) Schematics of canonical N pathway activation and of the reporter assays. (i) Cleavage of N induced by Delta binding results in the cytoplasmic domain of N (NICD), in association with its transcriptional effector Su(H), entering the nucleus, and in conjunction with the co-activator Mastermind (Mam) activating transcription of genes with Su(H) binding sites. (ii) and (iii) N reporter proteins in which the intracellular domain of N (NICD) is replaced with either GAL4-VP16 (NGV, ii) or LexA-VP16 (NLV, iii) were expressed under the control of the α-tubulin promoter, which is active in most or all cells. Endogenous Delta binding results in cleavage of the proteins, releasing GAL4-VP16 and LexA-VP16 from the membrane, allowing them to enter the nucleus and activate transcription of UAS.dGFP and LexOP.dGFP reporters. dGFP encodes destabilized GFP. (C) An antennal lobe showing N reporter activity in ORNs that project to glomerulus VA6 following chronic exposure to geranyl acetate. The antennal lobe was stained with anti-Bruchpilot antibody (magenta) that labels presynaptic active zones [109]. N reporter activity was detected with anti-GFP antibody (green). The circuit diagrams presented here and in subsequent figures were adapted from Wilson [10].

Flies detect odorants via the activation of one or more of approximately 50 distinct subpopulations of olfactory receptor neurons (ORNs) that decorate the antenna and maxillary palps. For the most part, each ORN subpopulation, defined by the expression of a single olfactory receptor, extends axons to a single, morphologically identifiable glomerulus, a neuropil compartment in the antennal lobe (Fig 1A and 1C). Here they synapse with the dendrites of second order projection neurons (PNs) that carry information to higher centers in the brain and with the dendrites of local interneurons (LNs) that innervate most glomeruli and mediate crosstalk between them ([7–9], reviewed in [10]).

In addition to its affect on behavior, chronic odor exposure also leads to changes in the activity of these neurons in both flies and honeybees and to changes in glomerular volume in flies, honeybees and mice. These changes correlate with the formation of long-term memory [1,5,11–14]. Sensory neurons had been thought to play a passive role in the formation and storage of long-term memory, simply acting as conduits to relay information from the environment to the central brain [15]. More recently, work in flies [16–18] and mice [19,20] point to a more active role for ORNs in olfactory plasticity.

We previously showed that, in response to chronic odor exposure, a Notch (N) reporter protein that assays canonical N signaling is activated in ORNs of adult *Drosophila* in an odorant specific fashion, demonstrating that in mature sensory neurons the N reporter responds to environmental inputs [21]. N is a transmembrane receptor of an evolutionarily conserved intercellular signaling pathway that is essential for the development of diverse tissues including the nervous system (reviewed in [22–24]). More recently N has also been reported to be involved in synaptic plasticity and memory [25–35], although this role for N in mammalian excitatory neurons is controversial [36,37]. In the canonical N pathway, upon binding its ligand, the transmembrane proteins Delta (Dl) or Serrate, N undergoes a series of proteolytic cleavages. This results in the cytoplasmic domain of N, in association with its transcriptional effector Suppressor of Hairless (Su(H)), entering the nucleus and activating transcription (reviewed in [38]). There are however less well understood non-canonical modes of N function that do not utilize all the components of the canonical pathway and involve interactions with other molecules [39–45]. It has been suggested that the non-canonical pathways evolutionarily predate canonical N signaling [46].

Here we show that endogenous N activity in adult *Drosophila* ORNs is required for changes in glomerular volume and neuronal activity that occur as a consequence of chronic odor exposure. Together with our previous work [21], this demonstrates that in conjunction with olfactory inputs, N in ORNs is required for structural and physiological plasticity of neurons in the olfactory circuit. We have identified PNs as being the principal source of Dl that controls N pathway activity in ORNs, thus delineating a circuit in which, in conjunction with odor, N activity in the periphery regulates the activity of neurons in the central brain and Dl in the central brain regulates N activity in the periphery. By modulating the components of the N pathway in synaptic partners of this well defined neural circuit in adult *Drosophila*, we show that in regulating morphological plasticity, N is functioning by both canonical and non-canonical mechanisms with Dl signaling controlling the output by shifting the balance between these two transduction mechanisms. By acting as a node in two different pathways, N in ORNs can regulate plasticity in response to changes in the environment.

## Results

### Odorant dependent activation of N in ORNs

To begin to address the role of N in the function of the adult *Drosophila* nervous system, we previously adapted an *in vivo* assay for the transmembrane cleavage of N that monitors canonical N pathway activation (Fig 1B) [21]. Using this assay, we showed that N is activated in adult *Drosophila* ORNs in response to long-term odor exposure. N activation in ORNs is dependent on the canonical N ligand Dl. It is odor specific and is dependent on cognate odorant receptors and on synaptic vesicle release by the ORNs in which the odorant receptors are active [21]. Interestingly, N is sometimes not activated in the same ORNs that respond directly to the odorant as assayed by electrophysiology, but instead in distinct and sometimes non-overlapping sets of ORNs, suggesting that N activation reflects a neural feedback following processing of olfactory input in the brain. However, to simplify analysis, we focus here on ORNs which show essentially a one-to-one correspondence between odorant reception and N activation. For example, geranyl acetate (GA) strongly activates Or82a expressing ORNs that project to glomerulus VA6 (VA6 ORNs) and weakly activates several other classes [8,47,48]. When flies are chronically exposed to GA, we detect N reporter activity in VA6 ORNs (Fig 1C).

### N in ORNs is required for the increase in volume that glomeruli undergo when flies are chronically exposed to odor

Odorant-dependent N activation, as revealed by our *in vivo* N reporter assay, initiates and decays over relatively long periods, beginning 6 to 12 hours after the onset of odor exposure, reaching near peak levels after 24 hours, and decaying to ~60% of peak levels after 24 hours following odorant removal [21]. Given these kinetics, we asked whether N plays a role in activity dependent plasticity in the adult *Drosophila* olfactory system. In both flies [5,11] and honeybees [13], it has been demonstrated that chronic odor exposure results in an odor specific increase in volume of glomeruli and this increase in volume correlates with the formation of long-term habituation in flies [1,5,11] and long-term memory in honeybees [12,13]. An increase in the size of olfactory glomeruli is also associated with long-term memory formation in mice [14]. Therefore, we tested the extent to which N in VA6 ORNs is required for the increase in volume of VA6 upon chronic exposure to GA.

**Fig 2:**
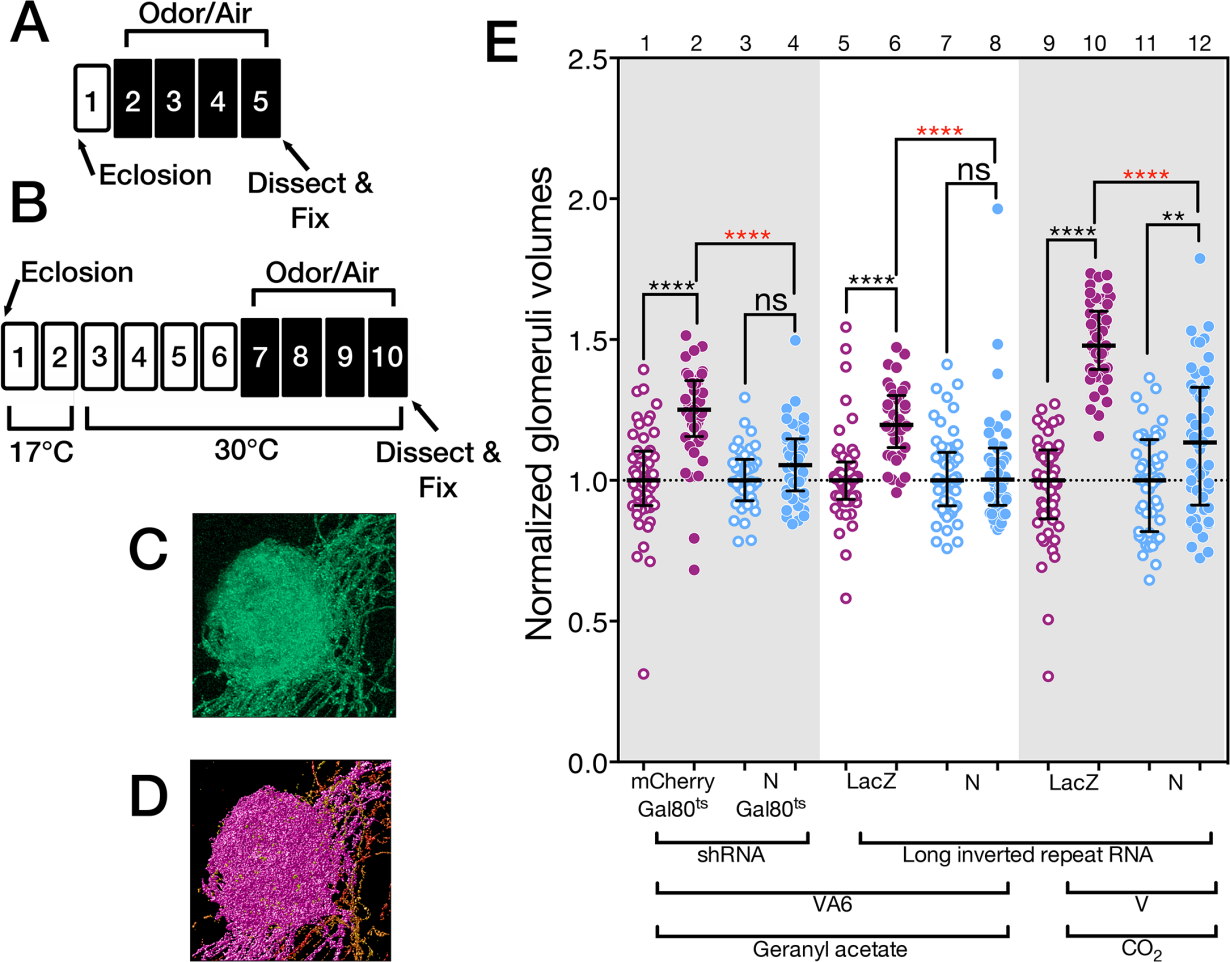
N regulates the increase in volume that glomeruli undergo when flies are chronically exposed to odor. (A & B) Schematics of experimental protocols. Here and in the remaining figures the numbers refer to days. White boxes indicate days prior to or following odor exposure. Black boxes indicate days of odor exposure. The protocol in B was used with flies carrying Gal80^ts^. (C) A maximum intensity projection of a deconvolved image stack of glomerulus VA6 detected by the expression of CD8-GFP. (D) 3-D reconstruction of C that was used to measure its volume (purple region). (E) The volumes of VA6 (lanes 1 to 8) or V (lanes 9 to 12) in female flies expressing the indicated transgenes in either VA6 ORNs under control of Or82a-GAL4 or in V ORNs under control of Gr21a-GAL4. All flies also carried UAS.CD8-GFP. The flies in lanes 1–4 carried tubP-gal80^ts10^. Here and in the figures below the data are displayed as scatter plots (each circle represents the volume of a single glomerulus) with median and interquartile ranges and were compared by Mann-Whitney tests. ns p>0.05; * p≤0.05; ** p≤0.01; *** p≤0.001; **** p≤0.0001. Blue scatter plots show data from N shRNA (lanes 3 & 4) or N long inverted repeat RNA (lanes 7, 8, 11 & 12). As controls, mCherry shRNA (lanes 1 & 2) or Lac Z long inverted repeat RNA (lanes 5, 6, 9 &10) were used and are shown in the purple scatter plots. Flies were exposed to either 1% GA in paraffin oil (lanes 2, 4, 6 and 8), paraffin oil (lanes 1, 3, 5 & 7), 5% CO_2_ (lanes 10 & 12), or air (lanes 9 & 11). Each pair of lanes represents air exposed flies (open circles) and the corresponding GA exposed flies (filled circles). The plot of each air exposed/odor exposed pair has been normalized to the median of the air exposed flies. Their median at 1 is indicated by the dashed line. This normalization allows us to compare the volumes of the odor exposed flies in each experiment. The uppermost, red, p-value refers this comparison. The black p-value compares air exposed flies with the corresponding GA exposed flies of the same genotype and is shown directly above each pair. The shRNAs are both inserted into attP2 and the long inverted repeat RNAs were outcrossed to cn bw flies for 6 generations.

We measured the VA6 glomerular volume by using Or82a-GAL4 [47] to drive expression of the membrane marker CD8-GFP in VA6 ORNs (Fig 2C and 2D) and co-expressed either control or *N* RNAi in these same ORNs to test the consequences of reducing N function. (In the course of this work we have used both shRNA and long inverted repeat RNAs. In the text we refer to both of these as RNAi. We specify which was used in the figure and figure legend for each experiment.) In our standard odor exposure paradigm (Fig 2A), flies are exposed to the odorant for four days on food and then assayed for VA6 glomerular size. Here and in figures below, the volumes of all the glomeruli were normalized to the median volume of the glomeruli of unexposed flies. The normalized median volume of the glomeruli of the unexposed flies is by definition 1 and is indicated by the dashed line in Fig 2E.

In flies expressing control RNAi, GA exposure results in an increase in the volume of VA6 (Fig 2E, lanes 5 & 6), which is reversible within 2 days when flies are removed from odor (S1 Fig). This increase in volume is abrogated in flies expressing *N* RNAi (Fig 2E, lanes 7 & 8). This difference is unlikely to be due to an effect of *N* RNAi on the development of VA6 ORNs, as the onset of Or gene expression in *Drosophila* occurs towards the end of pupal development, after ORN axons innervate the glomeruli, and is one of the final events in ORN differentiation ([49], reviewed in [50]). Nevertheless, to ensure that the effect on structural plasticity does not reflect a development defect, we expressed a temperature sensitive version of GAL80 that blocks transcriptional activation by Gal4 at 17°C, but permits it at 30°C [51]. Flies were raised to adulthood at 17°C. Two days after eclosion, they were shifted to 30°C for 4 days, prior to being exposed for 4 days at 30°C to GA or air (Fig 2B). Expression of *N* RNAi, but not control RNAi, in the adult VA6 ORNs of such flies also blocked the increase in volume of VA6 that occurs as a consequence of chronic exposure to GA (Fig 2E, lanes 1–4).

Similarly to GA, CO_2_ stimulates a single class of ORNs, which express the CO_2_ responsive Gr21a receptor [52,53]. We previously showed [21] that chronic exposure to CO_2_ results in N reporter activation in Gr21a ORNs, which project to the V glomerulus [48]. To assess whether N in V ORNs is required for the increase in volume of V that occurs as a consequence of long-term exposure to CO_2_ [5], we exposed flies expressing CD8-GFP and either control RNAi or *N* RNAi in V ORNs to CO_2_. As can be seen in Fig 2E, lanes 9–12, expression of *N* RNAi in V ORNs significantly reduced the extent of the CO_2_ induced volume increase.

Thus, we have demonstrated that endogenous N is required in the GA and CO_2_ responsive ORNs for the distinctive changes in brain structure that result from chronic exposure to each odorant.

### N activity in ORNs during chronic odor exposure alters odorant induced calcium flux in ORNs and PNs

**Fig 3:**
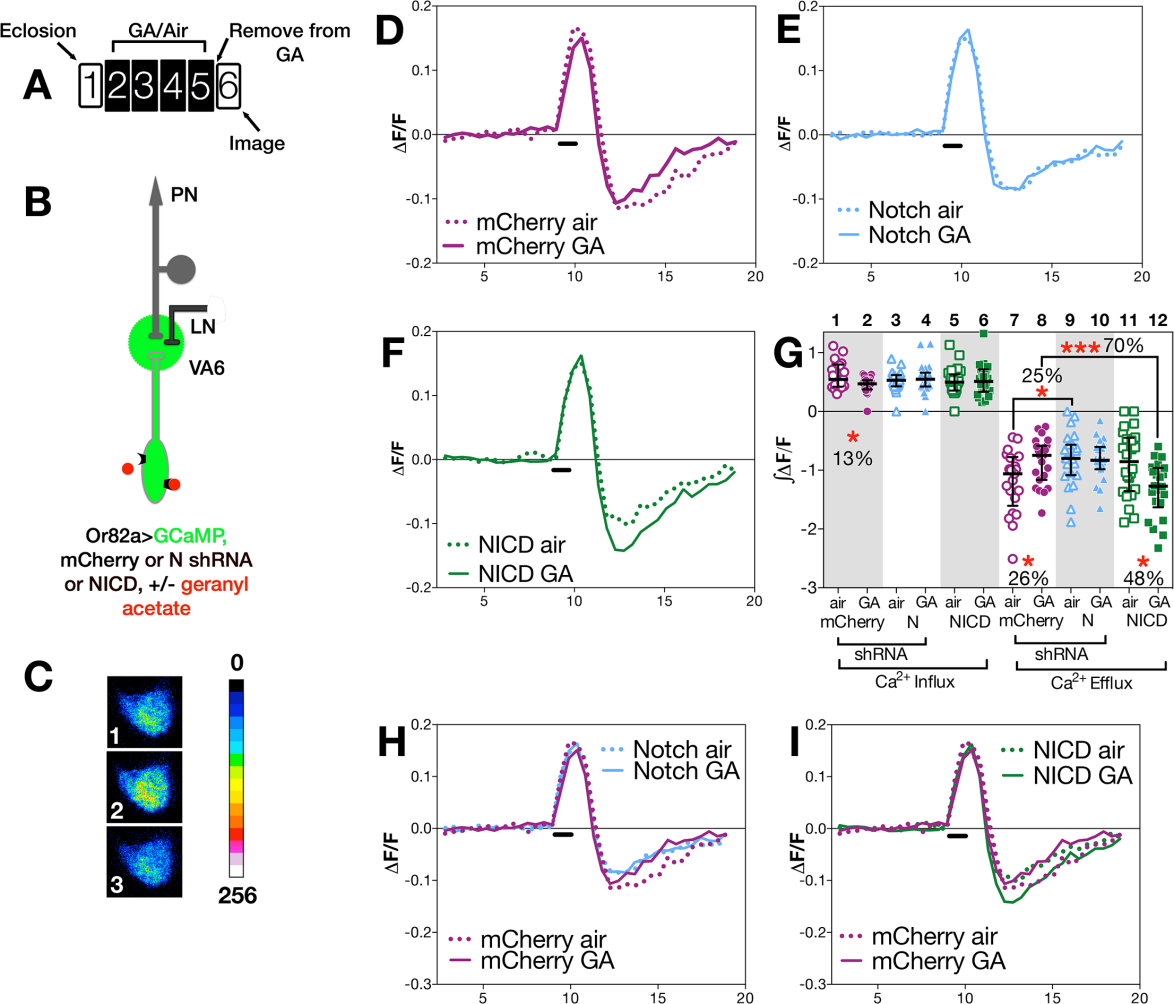
N activity in ORNs modulates ORN neuronal plasticity. (A) Schematic of experimental paradigm. Here and in Fig 4, female flies were exposed to 1% GA in paraffin oil or paraffin oil alone and removed from odor for one day prior to imaging. We assayed approximately 20 glomeruli for each combination of odor exposure and N activity. The traces (D-F, H,I) depict the median ΔF/F for each genotype and condition over time (in seconds on the X-axis). The black bar indicates the time of GA application; solid lines GA exposed flies; dotted lines air exposed flies. In D-I (as illustrated in B) flies were Or82a-GAL4; UAS.GCaMP3 carrying either (D) UAS.mCherry shRNA (control), (E) UAS.N shRNA (N RNAi) or (F) UAS.NICD (NICD). In H & I traces of ΔF/F for mCherry and N shRNA (H) or mCherry and NICD (I) are depicted in the same plot. (C) Representative pseudocolored images of VA6 ORNs prior to (1), during (2) and following (3) a 1.5 second pulse of GA. (G) ∫ΔF/F of calcium influx and efflux. The percentage change as well as the statistical significance of the change are depicted. Non significant p-values are not depicted. p-values were determined for GA exposed versus air exposed flies by comparing the areas under the influx and efflux peaks for each glomerulus.

**Fig 4:**
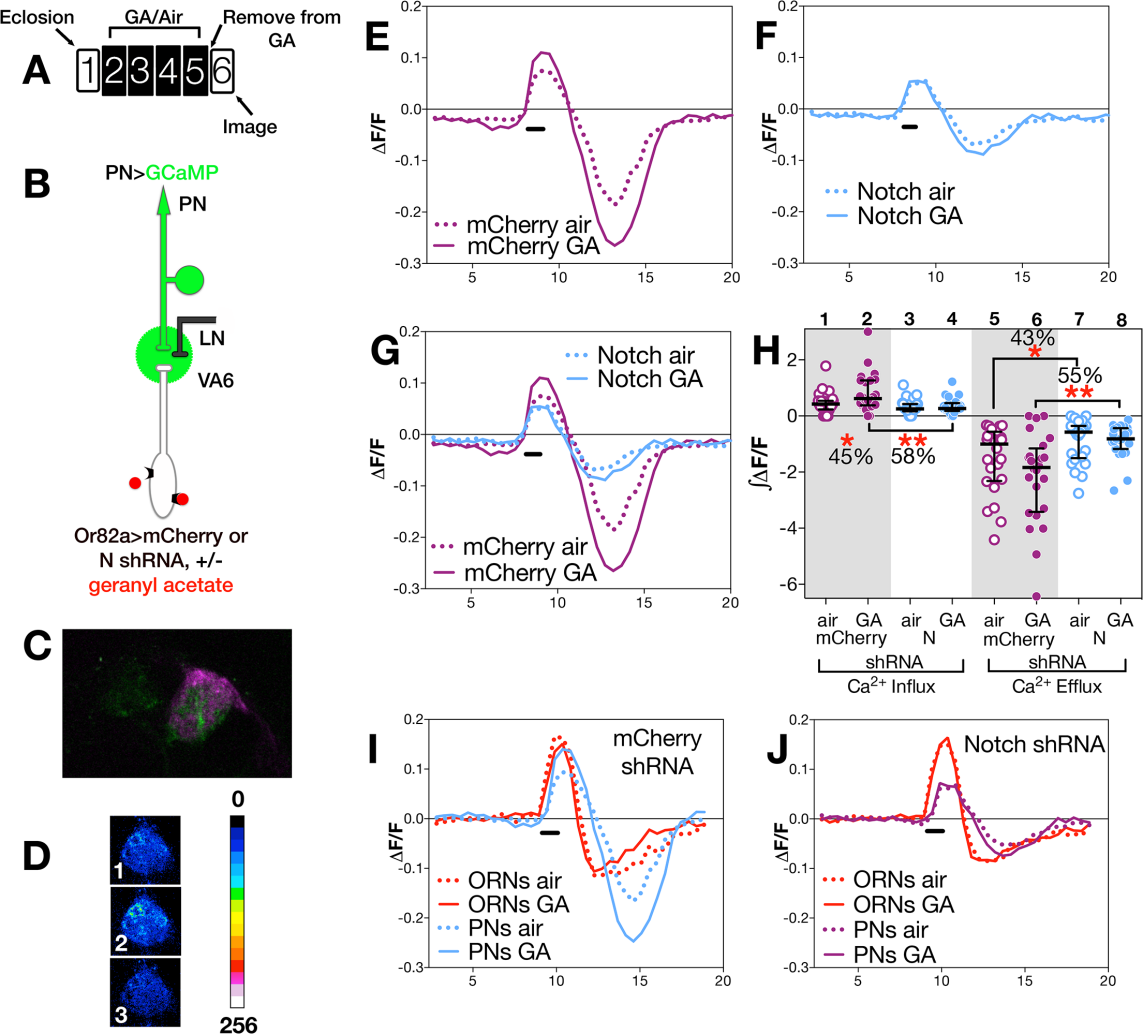
N in ORNs is required for PN neuronal plasticity. (A) Schematic of experimental paradigm. The traces (E-G, I,J) depict the median ΔF/F for each genotype and condition over time (in seconds on the X-axis). The black bar indicates the time of GA application; solid lines GA exposed flies; dotted lines air exposed flies. In E-H (as illustrated in B) flies were MZ612-GAL4 UAS.GCaMP6s/Or82a-LexAGAD; UAS.GCaMP6s LexOP.dsRED carrying either (E) LexOP.mCherry shRNA (control) or (F) LexOP.N shRNA (N RNAi). (C) Fluorescent image of VA6. MZ612-GAL4 driven GCaMP fluorescence is in green. Or82a-LexAGAD driven dsRED is in magenta and was used to identify VA6. (D) Representative pseudocolored image of VA6 PNs prior to (1), during (2) and following (3) a 1.5 second pulse of GA. (H) ∫ΔF/F of calcium influx and efflux. The percentage change as well as the statistical significance of the change are depicted. Non significant p-values are not depicted. p-values were determined for GA exposed versus air exposed flies by comparing the areas under the influx and efflux peaks for each glomerulus. In I & J traces of ΔF/F for ORNs and PNs of control (I) and N RNAi (J) flies are depicted in the same plot.

Long-term exposure to odors has been shown to alter the activity of neurons in the olfactory circuit in response to subsequent presentation of the same odor [4,5,11,13]. To assess whether N activity in ORNs influences this exposure dependent change in neural function, we subjected flies to our standard regimen of 4 days of exposure to air or GA on food, allowed them to recover in the absence of odor for one day (as diagrammed in Figs 3A and 4A), and then used calcium imaging to monitor odor evoked calcium activity in VA6 ORN axons and the activation of VA6 PNs following transient presentation of GA.

#### (i) Changes in ORNs

To assay odor evoked calcium activity in ORN axons we expressed the calcium sensor GCaMP3 [54] together with either control (*mCherry* shRNA) RNAi or *N* (shRNA) RNAi in VA6 ORNs, and used 2-photon microscopy to measure calcium responses in ORN axon terminals (Fig 3B). Representative pseudocolored images of VA6 ORNs from air exposed *mCherry* flies prior to, during, and following presentation of GA are shown in Fig 3C. Traces of the median fluorescent change over time (ΔF/F) for air and GA exposed flies that expressed *mCherry* RNAi are shown in Fig 3D, and those for air and GA exposed flies that expressed *N* RNAi are shown in Fig 3E. The ∫ΔF/F of calcium influx and efflux of air versus GA exposed flies expressing either *mCherry* or *N* RNAi are plotted in Fig 3G. In comparison to air exposed *mCherry* RNAi flies, GA exposed *mCherry* RNAi flies exhibited a small (13%) but statistically significant decrease in calcium influx into ORN axon terminals in response to transient exposure to GA (Fig 3D and 3G lanes 1 & 2). ORNs are noisy and fire in the absence of odor, and depending on the class of ORN, following odor offset, ORN activity can be suppressed below spontaneous levels (reviewed in [10]). This can be seen for air exposed *mCherry* RNAi flies by comparing Fig 3C panels 1 and 3, and in the traces in Fig 3D. In *mCherry* RNAi flies that had been chronically exposed to GA there was a statistically significant decrease (26%) in this calcium efflux (Fig 3D and 3G lanes 7 & 8).

In contrast to *mCherry* RNAi flies, we observe no significant difference in either calcium influx or efflux in air versus GA exposed *N* RNAi flies in response to transient presentation of GA (Fig 3E and 3G, lanes 3,4,9,10). Hence, reducing N function in VA6 ORNs appears to eliminate the decrease in both the initial influx as well as the subsequent efflux of calcium that results from prolonged GA exposure.

We next compared the effect of gain, rather than loss, of N function in VA6 ORNs on the response of flies that had been chronically exposed to GA to subsequent GA exposure. To do so, we expressed the N intracellular domain (NICD), a form of N that because it is no longer tethered to the membrane results in constitutive activity of the canonical N signal transduction pathway. As was the case for *N* RNAi expressing flies, there was no difference between air and GA exposed flies in calcium influx (Fig 3F and 3G, lanes 5 & 6). However, in contrast to *mCherry* RNAi expressing flies, where prior GA exposure resulted in decreased calcium efflux (Fig 3D), and to *N* RNAi flies where there was no difference in calcium efflux (Fig 3E), NICD flies that had been chronically exposed to GA show a statistically significant (48%) increase in calcium efflux (hyperpolarization) following odor offset (Fig 3F and 3G, lanes 11 & 12). Thus, gain as well as loss of N transducing activity in ORNs alters the effect of prior exposure to GA on the subsequent physiological response of ORNs to transient GA exposure. The effects of such alterations in N transducing activity on the physiological plasticity of ORNs are summed in Fig 3H and 3I, in which the traces of ΔF/F following prolonged GA versus air exposure are shown for the comparison between *mCherry* and *N* RNAi flies (Fig 3H), and for *mCherry* RNAi and NICD flies (Fig 3I).

#### (ii) Changes in PNs

To assay calcium responses in PNs, we expressed GCaMP6s [55] in VA6 PNs, and either control (*mCherrry*) or *N* RNAi together with dsRED in VA6 ORNs. 2-photon microscopy was used to measure calcium responses in PN dendrites (Fig 4B). A fluorescent image of VA6 with GCaMP in VA6 PNs and dsRED in VA6 ORNs is shown in Fig 4C, and representative pseudocolored images of VA6 PNs from air exposed *mCherry* flies prior to, during, and following presentation of GA are shown in Fig 4D. As can be seen in the traces of ΔF/F (Fig 4E) and the plots of ∫ΔF/F (Fig 4H, lanes 1 & 2), flies expressing *mCherry* RNAi in ORNs that had been chronically exposed to GA show a statistically significant increase (45%) in the amplitude of calcium activity in PNs in response to subsequent presentation of GA compared to air exposed *mCherry* RNAi expressing controls. This difference is abolished in GA exposed flies expressing *N* RNAi in VA6 ORNs (Fig 4F and 4H lanes 3 & 4). That modulating N activity in ORNs affects the physiological plasticity of PNs can be visualized in Fig 4G, where the traces of ΔF/F for *mCherrry* and *N* RNAi flies, shown in Fig 4E and 4F, are superimposed.

The dramatic effect of removing N from ORNs on the physiology of ORNS and PNs can be seen by comparing the calcium fluxes of ORNS and PNs of *mCherry* flies (Fig 4I) with those of *N* RNAi flies (Fig 4J). Importantly, we note that in the absence of prior GA exposure (i.e. in air exposed flies), we do not detect any significant difference between flies expressing *mCherry* or *N* RNAi in calcium influx into ORN axons (Fig 3H and 3G lanes 1,3) or PN dendrites (Fig 4G and 4H lanes 1,3). This suggests that expression of *N* RNAi in VA6 ORNs has no effect on the ability of ORNs or PNs from air exposed flies to respond to GA, but rather, as we observed in our volume experiments, that N is required in VA6 ORNs for plasticity of neurons in response to chronic odor exposure.

### Dl in PNs activates N in ORNs

In the canonical N pathway, N is activated by membrane tethered ligands found on neighboring cells. We previously showed that activation of our N reporter is dependent upon endogenous Dl. In the olfactory system, ORNs have significant contact with other ORNs, PNs, LNs and glia. To better define the circuit whereby odor exposure results in N activation in ORNs, we sought to identify the source of Dl that activates the N reporter in ORNs. We used appropriate Gal4 drivers to selectively manipulate Dl signaling capacity in ORNs, PNs, LNs or glia likely to abut a chosen ORN population, and monitored N activity using our N-LexA-VP16 reporter assay (Fig 1B; measured as the level of GFP fluorescence in the glomerulus innervated by the chosen ORN). We focused in particular on VA6 ORNs, which respond to GA and on ORNs that respond to ethyl butyrate (EB) and project to glomerulus VM2 (VM2 ORNs).

**Fig 5:**
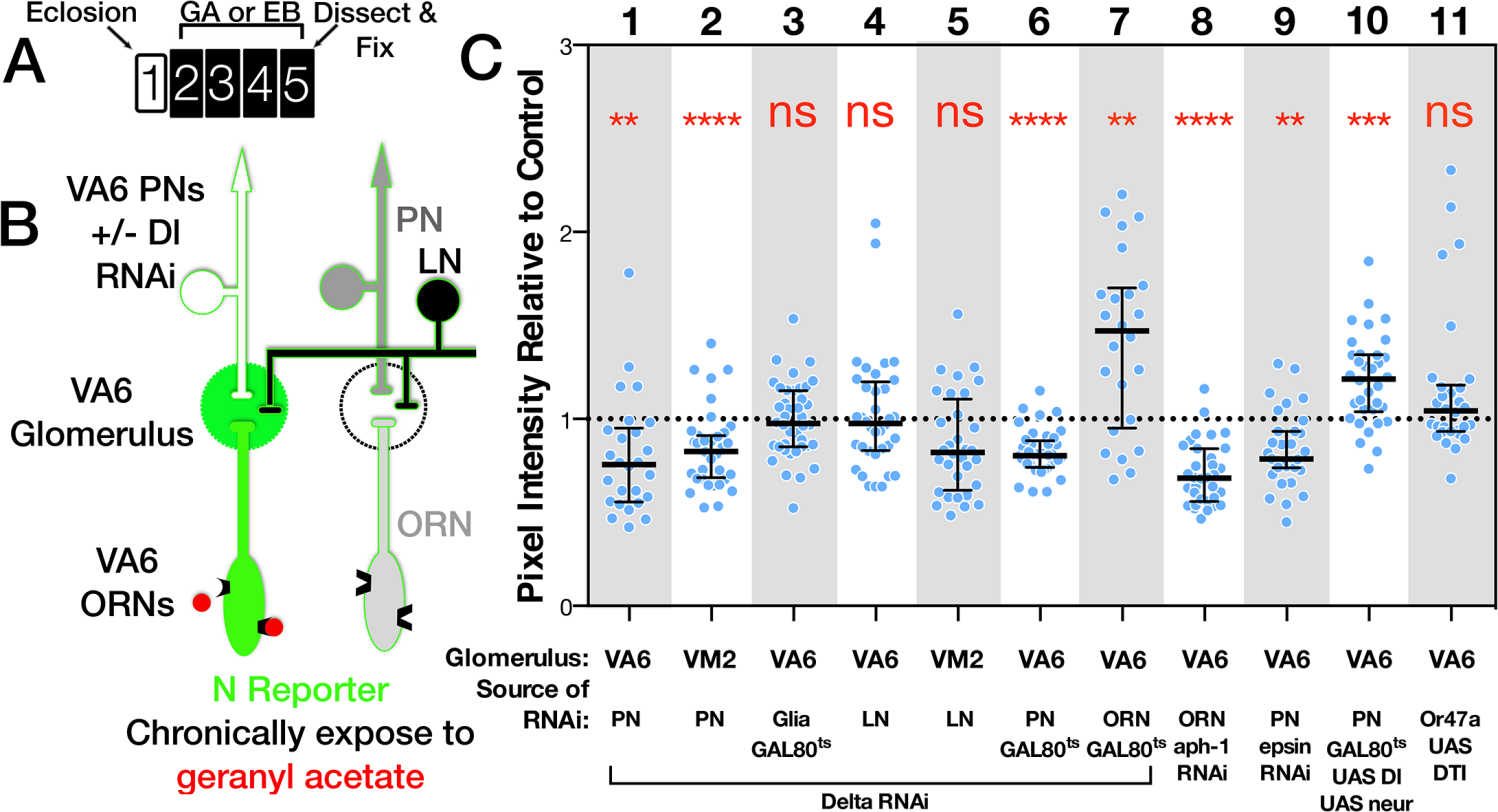
The source of the ligand for N reporter activity in ORNs is PNs. (A) Schematic of experimental protocol. One day old females were exposed to GA, EB or oil for 4 days and then analyzed. The protocol for flies carrying GAL80^ts^ was as in Fig 2B. Flies expressing UAS.DTI [56] (lane 11) were aged for one week prior to odor exposure. It has been shown that cell bodies and axons of ORNs expressing DTI are undetectable 5 days after eclosion [49]. (B) Diagram of the olfactory circuit depicting N reporter activation in VA6 ORNs in response to exposure to GA. Dl is being removed from VA6 PNs. C. N-LV LexOP.dGFP females carrying either UAS.Dl shRNA (lanes 1–5), UAS.DlXK8 long inverted repeat RNA (lanes 6 & 7), UAS.aph-1 shRNA (lane 8), UAS.Dl and UAS.neur (lane 10), UAS.epsin RNAi (lane 9), or UAS.DTI [56] (lane 11) in the indicated cell type were exposed to a 1:100 dilution of GA (VA6), EB (VM2) or oil for 4 days. In lanes 3, 6, 7 & 10 the flies also carried tubP-gal80^ts^ to limit expression to adults, and in lanes 6, 7 & 9 the flies also carried UAS.dicer-2 (dcr) to potentiate the RNAi. The level of N reporter activity seen in odor exposed flies carrying the indicated transgenes was normalized to the N reporter activity seen in odor exposed controls. The normalized activity of the odor exposed flies is presented. The entire data set comprising both odor exposed and air exposed flies is presented in S2 Fig. The following GAL4 drivers were used: VA6 PNs, MZ612; VM2 PNs, NP5103; VA6 glia, repo; VA6 and VM2 LNs, NP2426; VA6 ORNs, Or82a-GAL4.

We used GAL4 driven RNAi to knockdown Dl in ORNs, PNs, LNs, or glia. Flies were exposed to GA, or EB for 4 days, and the level of N reporter activity in VA6 or VM2 glomeruli in *Dl* RNAi expressing flies was determined relative to that of control RNAi (*mCherry*) expressing flies. (Fig 5; for each GAL4 driver, reporter activity of all the glomeruli was normalized to the median reporter activity of *mCherry* expressing flies. The normalized data is presented in the figure. The entire data set including the plots of the mCherry expressing flies is depicted in S2 Fig) RNAi knockdown of Dl in PNs that project to VA6 (illustrated in Fig 5B) or VM2 decreased the activity of the N reporter, respectively, in VA6 (Fig 5C lane 1) or VM2 (Fig 5C lane 2) glomeruli. In contrast, knockdown of Dl in LNs (Fig 5C lanes 4 & 5) or glia (Fig 5C lane 3) had no effect. The effect of reducing Dl in PNs is not a consequence of reducing Dl during development, because N reporter activity is still reduced when a temperature sensitive version of GAL80 was used to restrict expression of the *Dl* RNAi knockdown to adulthood (Fig 5C lane 6).

Each ORN is typically found in a sensilla with another class of ORN (reviewed in [10]). To ask whether these other ORNs might be a source of Dl, we used Or47a-GAL4 [47] driven diphtheria toxin (DTI) [56] to kill the other ORN housed in the same sensilla as VA6 ORNs. This had no effect on N reporter activity in VA6 ORNs (Fig 5C lane 11). Dl can also interact with N in cis, on the same cell, which inhibits N activation (reviewed in [57]). Accordingly, we found that expressing *Dl* RNAi in VA6 ORNs increased reporter activity in VA6 ORNs (Fig 5C lane 7). Together, with the positive results of Dl knockdown in PNs, and the negative results in LNs and glia, these results point to PNs as being the principal source of Dl that activates N in ORNs.

We then performed the converse experiment, raising the levels of Dl and Neuralized (Neur, a ubiquitin ligase required for Dl activity [58–60]) in adult VA6 PNs. This results, as expected, in an increase in reporter activity in VA6 (Fig 5C lane 10). None of the manipulations of Dl in VA6 PNs affected the projection of the PNs to VA6 (S3 Fig).

Odor dependent activation of the N reporter in VA6 ORNs is also dependent on Epsin and Aph-1, two other components of the canonical N pathway. Epsin mediates a select endocytic pathway required in “sending” cells for Dl signaling activity [61,62], whereas Aph1 is an essential component of γ-secretase required in “receiving” cells for Dl dependent transmembrane cleavage of N [63,64]. RNAi knockdown of Aph-1 in VA6 ORNs and of Epsin in VA6 PNs both decreased N reporter activity (Fig 5C, lanes 8,9). We note that Epsin-dependent Dl signaling activity may depend on the generation of mechanical tension across the intercellular ligand/receptor bridge that forms between Dl and N [38]. If so, activation of N in VA6 ORNs might require such tension to be generated across the synapses formed with VA6 PNs.

Taken in conjunction with our previous results showing that N activation in ORNs requires odorant receptor activation and synaptic vesicle release by these ORNs, these data define a circuit of activation of the canonical N pathway whereby synaptic transmission by ORNs activates Dl signaling in the PNs that synapse with these ORNs, and this Dl signal feeds back to promote N activation in the ORNs.

### Notch acts via both canonical and non-canonical pathways to regulate glomerular volume

**Fig 6:**
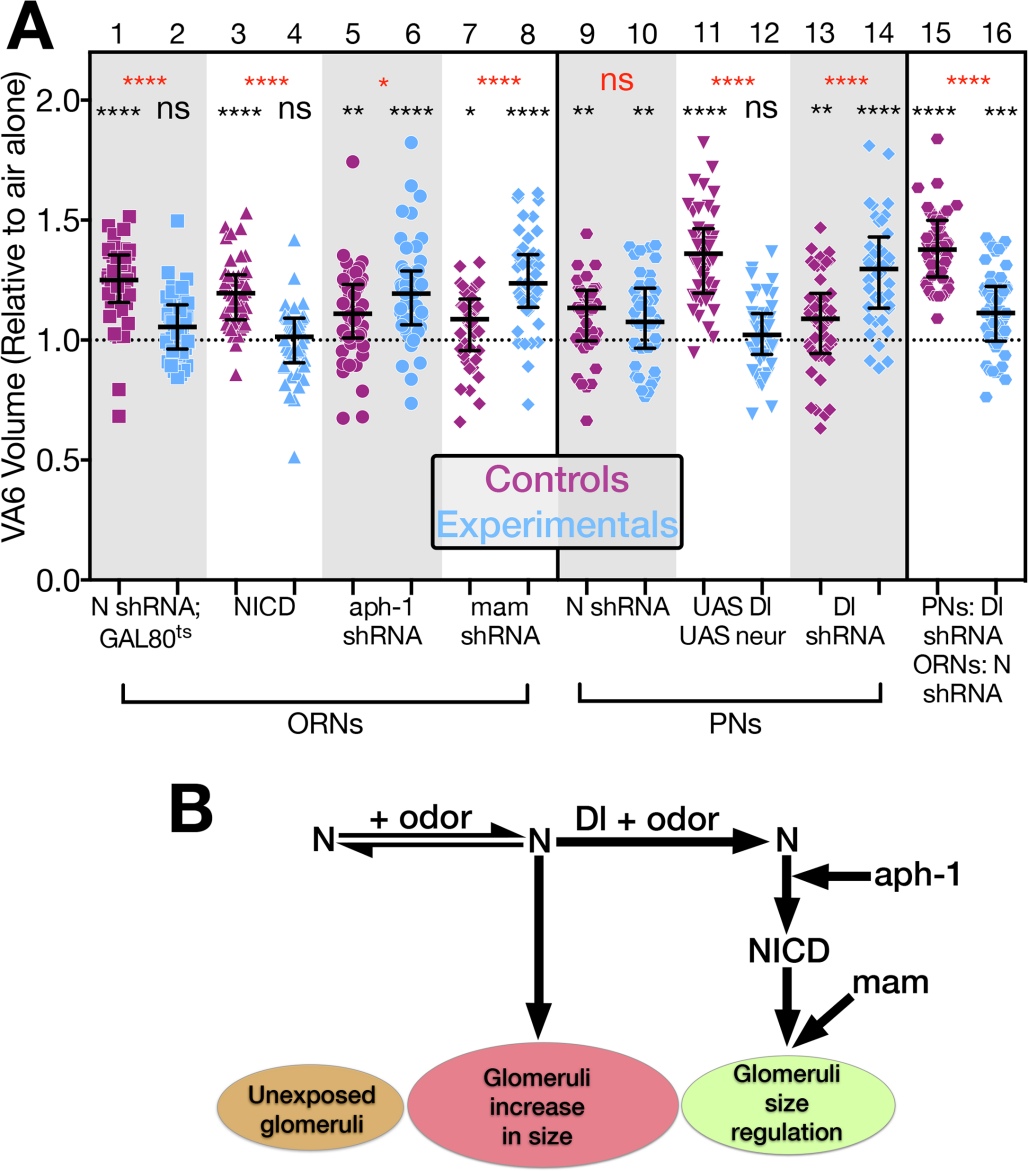
N regulates the increase in volume that glomeruli undergo when flies are chronically exposed to odor by both canonical and non-canonical mechanisms. (A) The volume of VA6 in GA exposed female flies expressing the indicated transgenes (blue) in either VA6 ORNs under control of Or82a-GAL4 (lanes 2, 4, 6, 8) or VA6 PNs under control of MZ612-GAL4 (lanes 10, 12, 14). With the exception of lane 11, the flies in the odd numbered lanes (purple) express control RNAs. The flies in lane 11 lacked the UAS constructs. Flies in lanes 1–8 carry UAS.cd8-GFP. Flies in lanes 9–14 carry Or82a.cd8-GFP. Flies in lanes 1 & 2 carry tubP-gal80^ts10^. Flies in lanes 15 & 16 express Dl RNAi in VA6 PNs under control of MZ612-GAL4, and LexAop.cd8-GFP together with either control (lane 15) or N RNAi (lane 16) in VA6 ORNs under control of Or82a-LexAGAD. The data have been normalized to the corresponding air exposed flies. Their median at 1 is indicated by the dashed line. Each pair of lanes represents GA exposed control flies (purple) and GA exposed mutant flies (blue). The uppermost, red, p-value refers to this comparison. The black p-value comparing air exposed with GA exposed flies is shown directly above each scatter plot. (Lanes 1 & 2 depict the same data as in Fig 2 lanes 2 & 4.) The results of these experiments are diagrammed in B which illustrates the interplay between the non-canonical and canonical N pathways in the regulation of glomerular volume. The entire data set of air exposed and GA exposed flies is presented in S4 Fig.

Our characterization of this circuit allowed us to manipulate components of the N pathway in synaptic partners and ask what effect these manipulations had on long-term odor induced neuronal plasticity. In the absence of Dl, N does not undergo the proteolytic cleavages required for its activation via the canonical pathway (Fig 1B). Therefore, loss of Dl in the PNs should have the same phenotype as the loss of N in ORNs, i.e. reducing Dl in VA6 PNs should block the increase in volume of VA6 in response to GA. To test this, we drove expression of control (*mCherry*) or *Dl* RNAi in VA6 PNs and used a direct fusion of CD8-GFP to the Or82a promoter (Or82a-CD8-GFP [48]) to measure the volume of VA6. Flies were exposed to air or GA as in Fig 2. As in Fig 2E, the volumes of all the glomeruli were normalized to the median volume of the glomeruli of air exposed flies. The normalized volumes of the GA exposed flies (controls in purple and experimentals in blue) are presented in Fig 6A. (The plots of the air exposed flies are not depicted in the figure. The entire data set is presented in S4 Fig). Whereas knockdown of N in VA6 ORNs (Fig 6A, lane 2) prevents the increase in glomerular volume that would otherwise result from prolonged GA exposure (Fig 6A, lane 1), Dl knockdown in VA6 PNs (Fig 6A, lane 14) fails to prevent this increase (Fig 6A, lane 13). Indeed, the increase in VA6 volume is larger in GA exposed flies that express *Dl* RNAi in PNs (Fig 6A, lane 14) than in flies that express *mCherry* RNAi in PNs (Fig 6A, lane 13). These data indicate that Dl in PNs is not required to activate N in ORNs to promote the volume increase, and that to promote the volume increase N is not acting via the canonical pathway. The fact that VA6 was larger in GA exposed flies lacking Dl than in GA exposed control flies indicates, rather, that the role of Dl in PNs is to limit the extent of the volume increase. These results raise the possibility that prolonged exposure to GA operates through a non-canonical N transduction pathway to promote the increase in VA6 volume, and that this output is constrained by Dl signaling from PNs, conceivably via activation of the canonical N pathway.

In the canonical pathway, in response to ligand binding, N is sequentially cleaved in its juxtamembrane and transmembrane domains (Fig 1B), and as shown in Fig 5C, lane 8, knockdown of Aph-1 in VA6 ORNs blocks GA dependent nuclear access of the N-LexA-VP16 reporter protein, indicating that it blocks canonical N signal transduction. However, in contrast to the RNAi knockdown of N (Fig 6A, lanes 1 & 2), Aph-1 knockdown does not block the GA dependent increase in VA6 volume (Fig 6A, lanes 5 & 6). This further indicates that N in ORNs is not promoting the increase in volume by the canonical N pathway. In fact, as was the case with reducing Dl in PNs, VA6 was larger in GA exposed flies lacking Aph-1 in VA6 ORNs (Fig 6A lane 6) than in GA exposed control (*mCherry*) flies (Fig 6A lane 5). It has been proposed that the more ancient mechanism of N function is non-canonical [46]. In this regard, it is interesting that in promoting this aspect of structural plasticity in the adult nervous system, N functions by a non-canonical mechanism.

That knocking down two components of the canonical N pathway, Dl in PNs and Aph-1 in ORNs, results in glomeruli that upon odor exposure are larger that those of control flies, suggests that while N is acting via a non-canonical pathway to promote the odor induced increase in volume, it acts via the canonical pathway to restrict the extent of the increase. To test this, we expressed, NICD in VA6 ORNs. This blocks the increase in volume of VA6 in response to exposure to GA (Fig 6A, lanes 3 & 4). Consistent with this, raising the levels of Dl and Neur in VA6 PNs (which by promoting N cleavage would increase the level of NICD) blocked VA6 enlargement upon chronic exposure to GA (Fig 6A, lanes 11 & 12).

The observation that VA6 was larger in GA exposed flies with reduced levels of Dl in PNs than in GA exposed control flies led us to hypothesize that the role of Dl in the regulation of glomerular volume is to switch the balance of N activity from the non-canonical to the canonical N pathway (Fig 6B). To eliminate the possibility that knocking down Dl in PNs was affecting glomerular volume by modulated the activity of N in PNs, we expressed N RNAi in VA6 PNs. This had no effect on the increase in volume of VA6 that occurs when flies are exposed to GA (Fig 6A lanes 9 & 10). We then asked whether in regulating glomerular volume Dl is acting via N in ORNs, as opposed to a non-N mechanism. To test this, we expressed *Dl* RNAi in VA6 PNs and either control or *N* RNAi in VA6 ORNs. As can be seen in Fig 6A lanes 15 and 16, in flies expressing *N* RNAi in VA6 ORNs (Fig 6A lane 16) there was a significant reduction in the extent of the GA induced volume increase of VA6 when compared to flies expressing control RNAi (Fig 6A lane 15), i.e. the phenotype is that of the loss of N in ORNs, not that of the loss of Dl in PNs. This indicates that Dl is regulating volume via N, and that one role of Dl is to shut off the non-canonical pathway.

Dl could be restricting the extent of the odor dependent increase in glomerular volume by simply shutting down the non-canonical N pathway. For example, if the non-canonical pathway involves a protein interacting with full length N at the membrane, Dl would terminate this interaction by promoting N cleavage. Alternatively, Dl could additionally be actively engaging the canonical N pathway (Fig 6B). This alternative is supported by our observation that expressing NICD, which results in constitutive canonical pathway N activity, in VA6 ORNs, blocks the increase in volume of VA6 in response to exposure to GA. Ideally, we would have liked to assay the requirement for the transcriptional effector Su(H). However, interpretation of Su(H) knockdown experiments is not straightforward. First, in the absence of N activation, Su(H) behaves as a transcriptional repressor [65,66]. Thus, in addition to preventing N mediated transcription, knocking down Su(H) would also result in inappropriate gene expression. Second, in the absence of Su(H), N protein is unstable [67,68]. Therefore, to assess if canonical N pathway components downstream of N processing are required for the Dl dependent restriction of glomerular volume, we used Or82a-GAL4 to express *mastermind* (*mam*) RNAi in VA6 ORNs. Because, Mam functions as a transcriptional co-activator for the N/Su(H) complex [69], knocking down Mam blocks transcription through the canonical pathway. As can be seen in Fig 6A, lanes 7 & 8, expressing *mam* RNAi in VA6 ORNs results in glomeruli that upon odor exposure are larger that those of flies expressing control RNAi, i.e. the phenotype resembles that of flies lacking Dl in PNs. With the caveat that there are N independent functions of Mam ([70,71], reviewed in [72]), this experiment suggests that removing N from the membrane is not sufficient to restrict the extent of the increase in volume of glomeruli that occurs upon chronic odor exposure, but rather this is an active process that requires transcriptional output of the canonical N pathway (Fig 6B).

## Discussion

**Fig 7:**
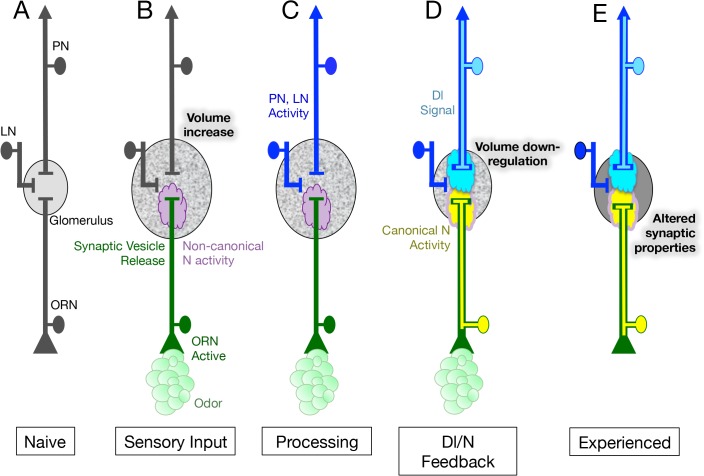
Model for the role of N in the regulation of glomeruli plasticity upon long-term odor exposure. (A) Neurons of the first olfactory synapse prior to odor exposure. (B) Chronic odor exposure induces synaptic vesicle release by ORNs as well as the activation of a non-canonical N pathway in ORNs that is required for an increase in glomerular volume. This non-canonical pathway does not require the N ligand Dl or Aph-1 dependent N cleavage, suggesting that in its non-canonical role N is acting at the membrane. (C) Neurotransmitter release by ORNs activates both PNs and LNs and in the simplest hypothesis activates Dl in the PNs. (D) Dl in PNs signals back to the ORNs, activating the canonical N signal transduction pathway to limit the increase in glomerular size. This feedback loop could be thought of as a homeostatic mechanism to regulate glomerular volume in the presence of chronic odor. (E) N activity in ORNs also alters the physiological response of ORNs to odor, resulting in changes in the activity of their cognate PNs. The change in ORN physiology persists for at least one day after exposure to chronic odor ceases.

ORNs receive input from the environment as well as from other neurons in the olfactory circuit. We previously showed that a N reporter protein is activated in adult *Drosophila* ORNs in response to environmental input and that its activity depends on and is modulated by other neurons in the olfactory circuit. In this paper we have presented evidence indicating that the N reporter reflects a role for endogenous N in olfactory plasticity. Specifically, we have shown that in adult *Drosophila*, N in ORNs mediates morphological (Fig 2) and physiological (Figs 3 and 4) plasticity in response to prolonged odor exposure. We have characterized a circuit whereby, in conjunction with odor, N activity in the periphery regulates the activity of neurons in the central brain and Dl in the central brain regulates N activity in the periphery (Fig 7). These results establish odorant dependent N activation as a physiologically relevant feedback circuit mediating structural and physiological responses to prolonged odor exposure.

We have shown that in regulating structural plasticity N acts by both canonical cleavage-dependent and non-canonical cleavage-independent mechanisms and present evidence suggesting that the role of Dl in the regulation of glomerular volume, is to switch the balance of N activity from the non-canonical to the canonical N pathway (Fig 6). In addition, we hypothesize that the activation of Dl in PNs, and the consequent activation of the canonical N pathway in ORNs, is controlled by synaptic transmission by ORNs (Fig 7C and 7D). We suggest that canonical and non-canonical N signaling occur simultaneously and are mutually exclusive for any one N molecule, with the balance between the two pathways determining the change in volume of the glomerulus in response to chronic odor exposure. This implies that in a single ORN, one protein functioning in two different pathways can regulate structural plasticity in response to changes in the environment (Fig 7B–7D).

### How does N regulate morphological plasticity?

Prior work has implicated N in the regulation of neuronal connectivity during development. For example, in mammalian primary neuron cultures and cell lines it has been shown that expression of NICD or activation of N by ligands on neighboring cells inhibits neurite outgrowth [73–76]. Similarly, in *Drosophila* it has been shown that expression of NICD inhibits arborization of atonal expressing axons that innervate the adult optic lobe [77]. Our observation that expressing NICD in adult ORNs or raising the levels of Dl and Neur in VA6 PNs blocks the increase in volume of VA6 in response to exposure to GA are in accord with these prior results. Non-canonical N signaling has also been shown to be involved in axon patterning in *Drosophila* [42,43]. It is conceivable that the mechanism by which N affects the structure of olfactory glomeruli in adult flies is analogous to these developmental processes.

In our experiments, we are modulating N activity in ORNs, and N acts cell autonomously. Yet, Sachse et al. found that the increase in volume of V upon long-term exposure to CO_2_ was not due to changes in the morphology of V ORNs and hypothesized that long-term odor exposure led to neuroanatomical changes in LNs [5]. This raises the possibility that N in ORNs modulates structural plasticity of glomeruli by regulating a neuromodulator or another signaling pathway that acts non-autonomously to affect the morphology of LNs. We discuss this further below. Alternatively, the increase in volume could be due to an odor and N dependent defasciculation of ORNs. This could result in changes in connectivity with PNs and LNs, and this change in connectivity could underlie the change in glomerular morphology that occurs upon chronic odor exposure.

### How does N regulate physiological plasticity?

Our results on the effect of long-term odor exposure on the physiology of ORNs and PNs differ from some previous studies. Whereas we observed that prior exposure to GA affected calcium flow into and out of VA6 ORNs upon subsequent presentation of GA (Fig 3D and 3G), Sachse et al. found that long-term exposure to CO_2_ had no effect on the physiology of Gr21a expressing ORNs [5]. Additionally, while we found that prior exposure to GA resulted in enhanced activation of VA6 PNs upon subsequent presentation of GA (Fig 4E and 4H), Das et al. observed that prior exposure to EB led to a reduction in EB evoked PN responses in EB responsive PNs [11]. Differences between our experimental paradigm and that of the previous studies could account for these disparities. For instance, unlike the earlier studies, we removed the flies from odor for one day prior to imaging. Additionally, CO_2_ and EB are repulsive to flies at the concentrations used [5,11,53], and prior exposure to CO_2_ or EB resulted in decreased behavioral responses to subsequent presentation of the odors [5,11], which might be expected to dampen PN responses. GA at the concentration we used is attractive to flies [78,79] (S5 Fig), and we observed that prior exposure to GA resulted in an increase in attraction to GA (S5 Fig). The flies were exposed to GA on food and following starvation showed an enhanced preference for GA (S5 Fig) We interpret the behavioral responses we observed as indicating that the flies are making an association between GA and food. This behavior is analogous to the “imaginal conditioning” described by Siddiqi and co-workers [6,18]. The enhanced attraction to GA might be expected to result in enhanced activation of VA6 PNs.

We have shown that knocking down N in ORNs affects activation of the downstream PNs (Fig 4E–4J). Cells postsynaptic to the N mutant ORN are all wild type, so the mutant physiological phenotypes must be propagated from the mutant ORN to the wild type cells. One parsimonious hypothesis for the altered PN activation observed in *N* RNAi flies is that it results from the altered responses observed in the ORNs. In control RNAi flies prior odor exposure results in a decrease in both calcium influx (depolarization) and the subsequent calcium efflux (hyperpolarization) compared to unexposed flies (Fig 3D and 3G). These changes do not occur in *N* RNAi flies (Fig 3E and 3G). Alternatively, an odor and N dependent change in ORN fasciculation, that results in changes in connectivity with PNs or LNs, could underlie the change in PN activation. A third, not necessarily exclusive, hypothesis is that N is regulating the expression of a neuromodulator secreted by ORNs that affects activation of postsynaptic neurons, both PNs and LNs. The *Drosophila* genome contains at least 42 genes that encode neuropeptides, peptide hormones or protein hormones [80–82] of which at least 16 appear to be expressed in adult antennae. Alternatively, as mentioned above, N in ORNs could be regulating another signaling pathway that acts non-autonomously.

Our data indicate that in response to chronic odor exposure, N regulates the volume of glomeruli by both non-canonical cleavage-independent and cleavage-dependent canonical N pathways. We do not know if in regulating physiological plasticity N is also acting by both canonical and non-canonical mechanisms.

### What role do PNs play in the regulation of N?

Because the N mediated increase in volume, in response to odor, does not require Dl in PNs (Fig 6), it is conceivable that the increase in volume is an autonomous property of ORNs and will occur in the absence of PN activation or of synaptic transmission by ORNs (Fig 7B).

We have shown here that activation of the canonical pathway is dependent on Dl in PNs (Fig 5). We showed previously that activation of the canonical pathway is dependent on synaptic vesicle release by ORNs [21]. It is possible that activation of PNs by their cognate ORNs promotes the ability of Dl to activate the canonical pathway. For instance, activation of PNs could lead to an increase in the amount of Dl at the the cell surface that is able to bind to N, or it could result in increased Neur and Epsin dependent endocytosis of Dl, which has been shown to a key requirement of canonical N pathway activation (reviewed in [38]). Alternatively, enhanced canonical N pathway activation could result from odor dependent changes in ORNs that are elicited in response to chronic odor exposure.

### What is the non-canonical pathway?

There is increasing evidence linking N to other signaling pathways (reviewed in [83]). An intriguing candidate for the non-canonical N pathway required for the odor induced increase in volume of glomeruli is the Abl tyrosine kinase pathway. The Abl pathway regulates actin dynamics which underlie changes in synaptic morphology and function [84–86]. Genetic and biochemical experiments have established a connection between N and the Abl pathway in both flies and mammals [41–43,87,88]. A component of the Abl pathway, the adaptor protein Disabled (Dab), has been shown to physically associate with N in fly heads and mammalian brains [43,89,90]. Supporting the involvement of the Abl pathway, we have found that both knocking down Dab with RNAi as well as elevating the levels of Dab in VA6 ORNs affected activation of the N reporter (S6 Fig). Over-expression of Dab also blocked the increase in volume of VA6 in flies chronically exposed to GA. In fact there was an odor dependent decrease in volume and an odor dependent change in the morphology of VA6 (S6 Fig).

Two alternate candidates for the non-canonical pathway are the TORC2 and Wingless/Wnt pathways. The TORC2 pathway has been shown to regulate actin polymerization, and is required for long-term memory in both mammals and flies [91]. In a model of neglect-induced apoptosis in mammalian cells, it has been shown that membrane associated N interacts functionally with TORC2 [45], and N activation of TORC2 has been shown to be required for the regulation of neural stem cells in both flies and mammals [44]. Wnts have been shown to be involved in the structural and functional plasticity of synapses, and Wingless has been shown to be required for long term memory in flies [92]. The activated form of the Wnt pathway component β-catenin has been shown to physically associate with membrane bound N in both flies [39] and mammalian embryonic stem cells [40].

### An active role for ORNs in olfactory plasticity

Recent work has attributed olfactory habituation in response to long-term odor exposure in Drosophila to a potentiation of inhibitory transmission between LNs and PNs (reviewed in [93]). Our work demonstrating that N is required in ORNs for changes in the morphology and physiology of neurons that occur as a consequence of chronic odor exposure reveals an additional aspect to the regulation of olfactory plasticity and highlights the importance of experience dependent plasticity at the first olfactory synapse.

## Materials and Methods

### Constructs and fly stocks

The following transgenic fly lines were generated: UAS.NICD: N^Intra1790^ [68] lacking the 3’ N UTR was subcloned into pValium20 [94]. Or82a-LexA-GAD: The Or82a promoter, amplified from genomic DNA using the primers AGGAGGCTGAGGATTACAAGGAACG and GACCCCAGTTTCTAGAACATGAAAGGATTG was subcloned into pBPnlsLexA—GADflUw [95] Addgene plasmid 26232. LexOP.mCherry shRNA and LexOP.N shRNA: The UAS sequences in UAS.mcherry and UAS.N shRNA TRiP.HMS00015 [94] were replaced with the LexA operator sequences of pJFRC19-13XLexAop2-IVS-myr::GFP [95] Addgene plasmid 26224. LexOP.dsRED: The myr::GFP in pJFRC19-13XLexAop2-IVS-myr::GFP [95] Addgene plasmid 26224 was replaced with DsRed2 from pENTR-DsRed2 N1 (CMB1) (Eric Campeau, Addgene plasmid # 22523). UAS.DlXK8 (long inverted repeat RNA): Sequences corresponding to nucleotides 221–500 of Dl mRNA GenBank ID X06289.1 were subcloned as an inverted repeat into pUdsGFP [96]. UAS.espin RNAi: Espin DNA, generated by RT-PCR with the primers GCAGCCAACCTTCGTTGGAGG and GGCTGCCATCATGAGCGAG, was subcloned as an inverted repeat into pUdsGFP [96].

Other transgenic stocks were Or82a-GAL4 [47], Or47a-GAL4 [47], Gr21a-GAL4 [52], UAS.mCD8-GFP [97], Or82a-CD8-GFP [48], 13XLexAop2-mCD8::GFP [95], UAS.Ni [98], UAS.N shRNA TRiP.HMS00015 [94], UAS.IRlacZ [99], UAS.Dl shRNA TRiP.HMS01309, UAS.aph-1 shRNA TRiP.HMS01693, UAS.mam long hairpin RNA TRiP.JF02881, UAS.mCherry in attP2 and attP40, 20XUAS.GCaMP3 in attP2 [100], 20XUAS.GCaMP6s in attP40 and VK00005 [55], N-LV [21], LexOP.dGFP [21], UAS.^myc^Dl [61], UAS.neur [59], UAS.DTI [56], NP5103 [101], repo-GAL4 [102], NP2426 [103], UAS-Dcr-2.D [104], tubP-gal80ts [51], UAS.Dab [105], UAS.Dab shRNA TRiP.HMS02482.

### Measurement of glomerular volume

Brains were dissected, fixed and stained as described by Pfeiffer et al. [95]. Glomeruli were labelled with the membrane marker mCD8-GFP which was detected with rabbit anti-GFP (Invitrogen), followed by DyLight 488 conjugated Donkey anti rabbit F(ab’)_2_ fragment (Jackson), and were mounted in Slowfade (Invitrogen).

Volume measurements were obtained from confocal Z stacks gathered using a Leica SP5 that were collected from images separated by 130nm. The same microscope settings were used throughout an experiment. The stacks were deconvolved using Huygens Essential Software version 4.1.1p1 64b for Windows 7 (Scientific Volume Imaging, The Netherlands. http://www.svi.nl/HuygensProducts) and the volume determined using Huygens object analyzer. When using Gal4 driven UAS.CD8-GFP to label glomeruli the high fluorescence levels allowed the default settings of the object analyzer to be used. For the weaker fluorescence that resulted from using CD8-GFP directly fused to the Or82a promotor, the settings were manually adjusted to obtain the best 3D reconstruction for volume measurement. Data was plotted in GraphPad Prism 6.0f for Mac OS X (GraphPad Software, San Diego California USA, www.graphpad.com) and analyzed by two-tailed Mann-Whitney tests. All experiments were carried out blind.

### 2-photon image analysis

Flies were prepared for 2-photon microscopy, odor was delivered, and imaging performed as described [106–108]. Images were acquired at 472 ms per frame with a resolution of 300 X 300 pixels. Odor was applied after 19 frames when imaging ORNs and after 31 frames when imaging PNs. Geranyl acetate was administered for 1.5 seconds from 100ml bottles containing 40ul of geranyl acetate on filter paper. Odor onset was controlled by TriggerSync software (Prairie Technologies) and solenoid valves (Parker Hannifin Corporation). The air flow rate was 500ml/minute. Bottles lacking geranyl acetate were used to confirm that the changes in fluorescence were not due to mechanical stimulation. The resulting T stacks were quantified by ImageJ using the Intensity V Time Monitor plugin (http://rsb.info.nih.gov/ij/plugins/dynamic-profiler/index.html), then imported into Prism. Because shutter noise caused a rise in calcium influx, the first 5 frames were omitted from further analysis. The average of the next 10 frames for ORNs or 15 frames for PNs were then used as the baseline to produce the fractional differences, (Value-Baseline)/Baseline, i.e. ∆F/F for each sample. ∫∆F/F was calculated from the area above and below the baseline following odor addition. Peaks less than 20% the minimum to maximum Y distance or comprising less than 4 adjacent points were omitted.

To assay calcium flux in ORNs the following number of samples were imaged: mCherry air, 22; mCherry GA, 23; N air, 23; N GA 22; NICD air, 24; NICD GA, 22. To assay calcium flux in PNs the following number of samples were imaged: mCherry air, 24; mCherry GA, 24; N air, 25; N GA, 23.

### Notch reporter assays

N reporter activity was determined as described previously [21].

## Supporting Information

S1 Fig The GA induced volume increase of VA6 is reversible. (A) Schematic of experimental protocol. One day old females expressing CD8-GFP and mCherry shRNA in VA6 ORNs under control of Or82a-GAL4 were exposed to a 1:100 dilution of GA or oil for 4 days. Flies were dissected immediately upon removal from odor (Day 0) or removed from odor and exposed to oil for 2 days prior to dissection (Day 0 + 2 days). (B) Scatter plots showing the volumes of VA6 glomeruli from air exposed (open circles) or GA exposed (filled circles) flies. Each circle represents the volume of a single glomerulus. The scatter plots are presented with median and interquartile ranges and were compared by Mann-Whitney tests. ns p>0.05; **** p≤0.0001. The volumes of all the glomeruli from each air/GA exposed pair were normalized to the median volume of the glomeruli of air exposed flies. The median volume of the glomeruli of the air exposed flies is by definition 1 and is indicated by the dashed line. This normalization allows us to compare the volumes of the GA exposed flies. The uppermost, red, p-value refers this comparison. The black p-value compares air exposed flies with the corresponding GA exposed flies and is shown directly above each pair.
(TIFF)

S2 Fig The source of the ligand for N reporter activity in ORNs is PNs. Scatter plots showing N reporter activity in the indicated glomeruli of odor exposed control flies (odd numbered lanes, purple) or flies expressing the indicated transgenes (even numbered lanes, blue) in either ORNs or PNs. Each circle represents the activity of a single glomerulus. The scatter plots are presented with median and interquartile ranges and were compared by Mann-Whitney tests. ns p>0.05; ** p≤0.01; *** p≤0.001; **** p≤0.0001. Each set of two lanes represents control flies and mutant flies from one experiment. For each GAL4 driver, reporter activity of all the glomeruli was normalized to the median reporter activity of the control flies. The median reporter activity of the control flies is by definition 1 and is indicated by the dashed line. The p-value for this comparison is indicated in red above each pair. N-LV LexOP.dGFP females carrying either UAS.Dl shRNA (lanes 2, 4, 6, 8 & 10), UAS.DlXK8 long inverted repeat RNA (lanes 12 & 14), UAS.aph-1 shRNA (lane 16), UAS.epsin RNAi (lane 18), UAS.Dl and UAS.neur (lane 20), or UAS.DTI [56] (lane 22) in the indicated cell type were exposed to a 1:100 dilution of GA (VA6), EB (VM2) or oil for 4 days. In lanes 5, 6, 11, 12, 13, 14, 19 and 20 the flies also carried tubP-gal80^ts^ to limit expression to adults, and in lanes 11–14, 17 and 18 the flies also carried UAS.dicer-2 (dcr) to potentiate the RNAi. The control flies in lanes 1, 3, 5, 7, 9 & 15 expressed mCherry shRNA which is inserted into the same chromosomal location as the indicated transgenes. In lanes 11, 13, 17, 19 & 21 the controls lacked the indicated RNAi (lanes 11, 13 & 17), or the indicated UAS constructs (lanes 19 & 21). The following GAL4 drivers were used: VA6 PNs, MZ612; VM2 PNs, NP5103; VA6 glia, repo; VA6 and VM2 LNs, NP2426; VA6 ORNs, Or82a-GAL4.
(TIF)

S3 Fig Manipulating the levels of Dl or N in VA6 PNs does not affect the projection of the PNs to VA6. Brains of flies expressing CD8-GFP and the indicated transgenes in VA6 PNs, under control of MZ612-GAL4, were stained with anti-GFP (green) and anti-Bruchpilot (Brp (nc82), magenta) antibodies. In all cases, CD8-GFP expression can be detected in VA6 indicating that expression of the transgenes does not affect projection of VA6 PNs to VA6. MZ612-GAL4 is not expressed exclusively in VA6 PNs.
(TIF)

S4 Fig N regulates the increase in volume that glomeruli undergo when flies are chronically exposed to odor by both canonical and non-canonical mechanisms. Scatter plots showing the volumes of VA6 glomeruli in control flies (purple) or flies expressing the indicated transgenes (blue) in either ORNs or PNs. Each symbol represents the volume of a single glomerulus. The scatter plots are presented with median and interquartile ranges and were compared by Mann-Whitney tests. ns p>0.05; * p≤0.05; ** p≤0.01; *** p≤0.001; **** p≤0.0001. Each set of four lanes represents control flies (purple) and mutant flies (blue) from one experiment. The flies in the odd numbered lanes (open symbols) were exposed to air, and the flies in the even numbered lanes (filled symbols) were chronically exposed to GA. The volumes of all the glomeruli from each air/GA exposed pair were normalized to the median volume of the glomeruli of air exposed flies. The median volume of the glomeruli of the air exposed flies is by definition 1 and is indicated by the dashed line. This normalization allows us to compare the volumes of the GA exposed flies in each experiment. The uppermost, red, p-value refers this comparison. The black p-value compares air exposed flies with the corresponding GA exposed flies of the same genotype and is shown directly above each pair. The indicated transgenes were expressed in either VA6 ORNs under control of Or82a-GAL4 (lanes 1–16) or VA6 PNs under control of MZ612-GAL4 (lanes 17–28). Flies in lanes 1–16 carry UAS.CD8-GFP. Flies in lanes 17–28 carry Or82a.CD8-GFP. Flies in lanes 1–4 carry tubP-gal80^ts10^. Flies in lanes 29–32 express Dl RNAi in VA6 PNs under control of MZ612-GAL4, and LexAop.cd8-GFP together with either control (lane 29 and 30) or NRNAi (lane 31 and 32) in VA6 ORNs under control of Or82a-LexAGAD. The controls are mCherry shRNA (lanes 1, 2, 5, 6, 9, 10, 13, 14, 17, 18, 25, 26, 29 and 30), which is inserted into the same chromosomal location as the indicated transgenes. In lane 21 & 22 the flies lacked the indicated UAS constructs. Lanes 1–4 depict the same data as in Fig 2 lanes 1–4.
(TIFF)

S5 Fig Prolonged exposure to GA enhances attraction to subsequent presentation of GA. (A) Flies are attracted to GA. Five day old female flies expressing mCherry RNAi in VA6 ORNs under control of Or82a-GAL4 were starved for 24 hours and then placed in mesh covered beakers in the dark, where they had a choice between scintillation vials with the indicated concentrations of GA in 0.1% triton or solvent alone. The flies were counted at 24 hrs. Performance Index (PI) is (GA—solvent)/(total flies). Each point represents the PI of a single beaker, with 40 flies per beaker. Here and in C the scatter plots are presented with median and interquartile ranges and were compared by Mann-Whitney tests. ** p≤0.01; *** p≤0.001; non-significant differences are not marked. Or82a-GAL4 flies were outcrossed to cn bw flies for 6 generations. (B & C) Prolonged exposure to GA enhances attraction to subsequent presentation of GA. One day old female flies expressing mCherry RNAi in VA6 ORNs under control of Or82a-GAL4 were exposed on food to 1% GA in paraffin oil (GA, closed circles) or to paraffin oil alone (air, open circles) for 4 days. The flies were removed from odor, starved for 24 hours, and then placed in mesh covered beakers in the dark where they had a choice between 2% ethyl butyrate or the indicated concentrations of GA, all diluted in 0.1% triton. The flies were counted at 24 hours. PI is (GA—EB)/(total flies). Each point represents the PI of a single beaker, with 40 flies per beaker. In the log plots the larger the number the greater the attraction to GA. The data were normalized by dividing by the median of the air exposed flies for each concentration of GA. ns, p>0.05; *, p≤0.05.
(TIF)

S6 Fig Disabled (Dab) affects N reporter activity and glomerular volume and morphology. One day old female flies were exposed to 1% GA in paraffin oil (GA) or paraffin oil alone (air) for 4 days, after which (A & B) N reporter activity or (C) VA6 size was determined. In A & B the level of N reporter activity seen in odor exposed flies carrying the indicated transgenes was normalized to the N reporter activity seen in odor exposed controls. In C the volumes of all the glomeruli from each air/GA exposed pair were normalized to the median volume of the glomeruli of air exposed flies. This normalization allows us to compare the volumes of the GA exposed flies in each experiment. The uppermost, red, p-value refers this comparison. The black p-value compares air exposed flies with the corresponding GA exposed flies of the same genotype and is shown directly above each pair. * p≤0.05; *** p≤0.001; **** p≤0.0001. Panels D-G illustrate the changes in morphology and size caused by over-expression of Dab in conjunction with odor. (A) Or82a-GAL4; NLV LexOP.dGFP with mCherry (control) or Dab shRNA. (B) Or82a-GAL4; NLV LexOP.dGFP without (control) or with UAS.Dab. (C-G) Or82a-GAL4; UAS cd8-GFP without (control) or with UAS.Dab.
(TIF)

## References

[1] Devaud JM, Acebes A, Ferrús A (2001) Odor exposure causes central adaptation and morphological changes in selected olfactory glomeruli in Drosophila. J Neurosci 21: 6274–6282. 11487650 10.1523/JNEUROSCI.21-16-06274.2001 PMC6763130

[2] Devaud JM, Acebes A, Ramaswami M, Ferrús A (2003) Structural and functional changes in the olfactory pathway of adult Drosophila take place at a critical age. J Neurobiol 56: 13–23. 10.1002/neu.10215 12767029

[3] Arenas A, Fernández VM, Farina WM (2009) Associative learning during early adulthood enhances later memory retention in honeybees. PLoS ONE 4: e8046 10.1371/journal.pone.0008046 19956575 PMC2779852

[4] Arenas A, Giurfa M, Farina WM, Sandoz JC (2009) Early olfactory experience modifies neural activity in the antennal lobe of a social insect at the adult stage. Eur J Neurosci 30: 1498–1508. 10.1111/j.1460-9568.2009.06940.x 19821839

[5] Sachse S, Rueckert E, Keller A, Okada R, Tanaka NK, et al (2007) Activity-dependent plasticity in an olfactory circuit. Neuron 56: 838–850. 10.1016/j.neuron.2007.10.035 18054860

[6] Chakraborty TS, Goswami SP, Siddiqi O (2009) Sensory correlates of imaginal conditioning in Drosophila melanogaster. J Neurogenet 23: 210–219. 10.1080/01677060802491559 19058083

[7] Gao Q, Yuan B, Chess A (2000) Convergent projections of Drosophila olfactory neurons to specific glomeruli in the antennal lobe. Nat Neurosci 3: 780–785. 10.1038/77680 10903570

[8] Hallem EA, Carlson JR (2006) Coding of odors by a receptor repertoire. Cell 125: 143–160. 10.1016/j.cell.2006.01.050 16615896

[9] Vosshall LB, Amrein H, Morozov PS, Rzhetsky A, Axel R (1999) A spatial map of olfactory receptor expression in the Drosophila antenna. Cell 96: 725–736. 10089887 10.1016/s0092-8674(00)80582-6

[10] Wilson RI (2013) Early Olfactory Processing in Drosophila: Mechanisms and Principles. Annu Rev Neurosci 36: 217–241. 10.1146/annurev-neuro-062111-150533 23841839 PMC3933953

[11] Das S, Sadanandappa MK, Dervan A, Larkin A, Lee JA, et al (2011) Plasticity of local GABAergic interneurons drives olfactory habituation. Proc Natl Acad Sci USA 108: E646–E654. 10.1073/pnas.1106411108 21795607 PMC3169145

[12] Hourcade B, Perisse E, Devaud JM, Sandoz J-C (2009) Long-term memory shapes the primary olfactory center of an insect brain. Learning & Memory 16: 607–615. 10.1101/lm.1445609 19794186

[13] Arenas A, Giurfa M, Sandoz JC, Hourcade B, Devaud JM, et al (2012) Early olfactory experience induces structural changes in the primary olfactory center of an insect brain. Eur J Neurosci 35: 682–690. 10.1111/j.1460-9568.2012.07999.x 22300014

[14] Jones SV, Choi DC, Davis M, Ressler KJ (2008) Learning-dependent structural plasticity in the adult olfactory pathway. J Neurosci 28: 13106–13111. 10.1523/JNEUROSCI.4465-08.2008 19052201 PMC2613972

[15] Davis RL (2004) Olfactory learning. Neuron 44: 31–48. 10.1016/j.neuron.2004.09.008 15450158

[16] Root CM, Masuyama K, Green DS, Enell LE, Nässel DR, et al (2008) A presynaptic gain control mechanism fine-tunes olfactory behavior. Neuron 59: 311–321. 10.1016/j.neuron.2008.07.003 18667158 PMC2539065

[17] Root CM, Ko KI, Jafari A, Wang JW (2011) Presynaptic facilitation by neuropeptide signaling mediates odor-driven food search. Cell 145: 133–144. 10.1016/j.cell.2011.02.008 21458672 PMC3073827

[18] Iyengar A, Chakraborty TS, Goswami SP, Wu C-F, Siddiqi O (2010) Post-eclosion odor experience modifies olfactory receptor neuron coding in Drosophila. Proc Natl Acad Sci USA 107: 9855–9860. 10.1073/pnas.1003856107 20448199 PMC2906832

[19] Kass MD, Rosenthal MC, Pottackal J, McGann JP (2013) Fear Learning Enhances Neural Responses to Threat-Predictive Sensory Stimuli. Science 342: 1389–1392. 10.1126/science.1244916 24337299 PMC4011636

[20] Abraham NM, Vincis R, Lagier S, Rodriguez I, Carleton A (2014) Long term functional plasticity of sensory inputs mediated by olfactory learning. eLife 3: e02109–e02109. 10.7554/eLife.02109.011 24642413 PMC3953949

[21] Lieber T, Kidd S, Struhl G (2011) DSL-Notch signaling in the Drosophila brain in response to olfactory stimulation. Neuron 69: 468–481. 10.1016/j.neuron.2010.12.015 21315258 PMC3216490

[22] Greenwald I (1998) LIN-12/Notch signaling: lessons from worms and flies. Genes Dev 12: 1751–1762. 9637676 10.1101/gad.12.12.1751

[23] Lai EC (2004) Notch signaling: control of cell communication and cell fate. Development 131: 965–973. 10.1242/dev.01074 14973298

[24] Louvi A, Artavanis-Tsakonas S (2006) Notch signalling in vertebrate neural development. Nat Rev Neurosci 7: 93–102. 10.1038/nrn1847 16429119

[25] Conboy L, Seymour CM, Monopoli MP, O'Sullivan NC, Murphy KJ, et al (2007) Notch signalling becomes transiently attenuated during long-term memory consolidation in adult Wistar rats. Neurobiol Learn Mem 88: 342–351. 10.1016/j.nlm.2007.04.006 17543552

[26] Costa RM, Honjo T, Silva AJ (2003) Learning and memory deficits in Notch mutant mice. Curr Biol 13: 1348–1354. 12906797 10.1016/s0960-9822(03)00492-5

[27] Dahlhaus M, Hermans JM, Van Woerden LH, Saiepour MH, Nakazawa K, et al (2008) Notch1 signaling in pyramidal neurons regulates synaptic connectivity and experience-dependent modifications of acuity in the visual cortex. J Neurosci 28: 10794–10802. 10.1523/JNEUROSCI.1348-08.2008 18945887 PMC6671381

[28] Ge X, Hannan F, Xie Z, Feng C, Tully T, et al (2004) Notch signaling in Drosophila long-term memory formation. Proc Natl Acad Sci USA 101: 10172–10176. 10.1073/pnas.0403497101 15220476 PMC454384

[29] Matsuno M, Horiuchi J, Tully T, Saitoe M (2009) The Drosophila cell adhesion molecule klingon is required for long-term memory formation and is regulated by Notch. Proc Natl Acad Sci USA 106: 310–315. 10.1073/pnas.0807665106 19104051 PMC2606903

[30] Presente A, Boyles RS, Serway CN, de Belle JS, Andres AJ (2004) Notch is required for long-term memory in Drosophila. Proc Natl Acad Sci USA 101: 1764–1768. 10.1073/pnas.0308259100 14752200 PMC341850

[31] Wang Y, Chan SL, Miele L, Yao PJ, Mackes J, et al (2004) Involvement of Notch signaling in hippocampal synaptic plasticity. Proc Natl Acad Sci USA 101: 9458–9462. 10.1073/pnas.0308126101 15190179 PMC438998

[32] Alberi L, Liu S, Wang Y, Badie R, Smith-Hicks C, et al (2011) Activity-induced Notch signaling in neurons requires Arc/Arg3.1 and is essential for synaptic plasticity in hippocampal networks. Neuron 69: 437–444. 10.1016/j.neuron.2011.01.004 21315255 PMC3056341

[33] Zhang J, Little CJ, Tremmel DM, Yin JCP, Wesley CS (2013) Notch-inducible hyperphosphorylated CREB and its ultradian oscillation in long-term memory formation. J Neurosci 33: 12825–12834. 10.1523/JNEUROSCI.0783-13.2013 23904617 PMC3728690

[34] Yoon K-J, Lee H-R, Jo YS, An K, Jung S-Y, et al (2012) Mind bomb-1 is an essential modulator of long-term memory and synaptic plasticity via the Notch signaling pathway. Mol Brain 5: 40 10.1186/1756-6606-5-40 23111145 PMC3541076

[35] Dias BG, Goodman JV, Ahluwalia R, Easton AE, Andero R, et al. (2014) Amygdala-Dependent Fear Memory Consolidation via miR-34a and Notch Signaling. Neuron: 1–13. 10.1016/j.neuron.2014.07.019 PMC4172484 25123309

[36] Zheng J, Watanabe H, Wines-Samuelson M, Zhao H, Gridley T, et al (2012) Conditional deletion of Notch1 and Notch2 genes in excitatory neurons of postnatal forebrain does not cause neurodegeneration or reduction of Notch mRNAs and proteins. J Biol Chem 287: 20356–20368. 10.1074/jbc.M112.349738 22505716 PMC3370217

[37] Sato C, Turkoz M, Dearborn JT, Wozniak DF, Kopan R, et al (2012) Loss of RBPj in postnatal excitatory neurons does not cause neurodegeneration or memory impairments in aged mice. PLoS ONE 7: e48180 10.1371/journal.pone.0048180 23110206 PMC3482205

[38] Kopan R, Ilagan MXG (2009) The canonical Notch signaling pathway: unfolding the activation mechanism. Cell 137: 216–233. 10.1016/j.cell.2009.03.045 19379690 PMC2827930

[39] Hayward P, Brennan K, Sanders P, Balayo T, DasGupta R, et al (2005) Notch modulates Wnt signalling by associating with Armadillo/beta-catenin and regulating its transcriptional activity. Development 132: 1819–1830. 10.1242/dev.01724 15772135 PMC2500123

[40] Kwon C, Cheng P, King IN, Andersen P, Shenje L, et al (2011) Notch post-translationally regulates β-catenin protein in stem and progenitor cells. Nature Cell Biology 13: 1244–1251. 10.1038/ncb2313 21841793 PMC3187850

[41] Song JK, Giniger E (2011) Noncanonical Notch function in motor axon guidance is mediated by Rac GTPase and the GEF1 domain of Trio. Dev Dyn 240: 324–332. 10.1002/dvdy.22525 21246649 PMC3070923

[42] Kuzina I, Song JK, Giniger E (2011) How Notch establishes longitudinal axon connections between successive segments of the Drosophila CNS. Development 138: 1839–1849. 10.1242/dev.062471 21447553 PMC3074455

[43] Le Gall M, De Mattei C, Giniger E (2008) Molecular separation of two signaling pathways for the receptor, Notch. Dev Biol 313: 556–567. 10.1016/j.ydbio.2007.10.030 18062953 PMC2262048

[44] Lee KS, Wu Z, Song Y, Mitra SS, Feroze AH, et al (2013) Roles of PINK1, mTORC2, and mitochondria in preserving brain tumor-forming stem cells in a noncanonical Notch signaling pathway. Genes Dev 27: 2642–2647. 10.1101/gad.225169.113 24352421 PMC3877754

[45] Perumalsamy LR, Nagala M, Banerjee P, Sarin A (2009) A hierarchical cascade activated by non-canonical Notch signaling and the mTOR-Rictor complex regulates neglect-induced death in mammalian cells. Cell Death Differ 16: 879–889. 10.1038/cdd.2009.20 19265851

[46] Layden MJ, Martindale MQ (2014) Non-canonical Notch signaling represents an ancestral mechanism to regulate neural differentiation. Evodevo 5: 30 10.1186/2041-9139-5-30 25705370 PMC4335385

[47] Fishilevich E, Vosshall LB (2005) Genetic and functional subdivision of the Drosophila antennal lobe. Curr Biol 15: 1548–1553. 10.1016/j.cub.2005.07.066 16139209

[48] Couto A, Alenius M, Dickson BJ (2005) Molecular, Anatomical, and Functional Organization of the Drosophila Olfactory System. Current Biology 15: 1535–1547. 10.1016/j.cub.2005.07.034 16139208

[49] Berdnik D, Chihara T, Couto A, Luo L (2006) Wiring stability of the adult Drosophila olfactory circuit after lesion. J Neurosci 26: 3367–3376. 10.1523/JNEUROSCI.4941-05.2006 16571743 PMC6673868

[50] Brochtrup A, Hummel T (2011) Olfactory map formation in the Drosophila brain: genetic specificity and neuronal variability. Curr Opin Neurobiol 21: 85–92. 10.1016/j.conb.2010.11.001 21112768

[51] McGuire SE, Le PT, Osborn AJ, Matsumoto K, Davis RL (2003) Spatiotemporal rescue of memory dysfunction in Drosophila. Science 302: 1765–1768. 10.1126/science.1089035 14657498

[52] Scott K, Brady R, Cravchik A, Morozov P, Rzhetsky A, et al (2001) A chemosensory gene family encoding candidate gustatory and olfactory receptors in Drosophila. Cell 104: 661–673. 10.1016/S0092-8674(02)02052-4 11257221

[53] Suh GSB, Wong AM, Hergarden AC, Wang JW, Simon AF, et al (2004) A single population of olfactory sensory neurons mediates an innate avoidance behaviour in Drosophila. Nature 431: 854–859. 10.1038/nature02980 15372051

[54] Tian L, Hires SA, Mao T, Huber D, Chiappe ME, et al (2009) Imaging neural activity in worms, flies and mice with improved GCaMP calcium indicators. Nat Meth 6: 875–881. 10.1038/nmeth.1398 PMC2858873 19898485

[55] Chen T-W, Wardill TJ, Sun Y, Pulver SR, Renninger SL, et al (2013) Ultrasensitive fluorescent proteins for imaging neuronal activity. Nature 499: 295–300. 10.1038/nature12354 23868258 PMC3777791

[56] Han DD, Stein D, Stevens LM (2000) Investigating the function of follicular subpopulations during Drosophila oogenesis through hormone-dependent enhancer-targeted cell ablation. Development 127: 573–583. 10631178 10.1242/dev.127.3.573

[57] del Álamo D, Rouault H, Schweisguth F (2011) Mechanism and significance of cis-inhibition in Notch signalling. Curr Biol 21: R40–R47. 10.1016/j.cub.2010.10.034 21215938

[58] Deblandre GA, Lai EC, Kintner C (2001) Xenopus neuralized is a ubiquitin ligase that interacts with XDelta1 and regulates Notch signaling. Dev Cell 1: 795–806. 11740941 10.1016/s1534-5807(01)00091-0

[59] Lai EC, Deblandre GA, Kintner C, Rubin GM (2001) Drosophila neuralized is a ubiquitin ligase that promotes the internalization and degradation of delta. Dev Cell 1: 783–794. 11740940 10.1016/s1534-5807(01)00092-2

[60] Pavlopoulos E, Pitsouli C, Klueg KM, Muskavitch MA, Moschonas NK, et al (2001) neuralized Encodes a peripheral membrane protein involved in delta signaling and endocytosis. Dev Cell 1: 807–816. 11740942 10.1016/s1534-5807(01)00093-4

[61] Wang W, Struhl G (2004) Drosophila Epsin mediates a select endocytic pathway that DSL ligands must enter to activate Notch. Development 131: 5367–5380. 10.1242/dev.01413 15469974

[62] Overstreet E (2004) Fat facets and Liquid facets promote Delta endocytosis and Delta signaling in the signaling cells. Development 131: 5355–5366. 10.1242/dev.01434 15469967

[63] Francis R, McGrath G, Zhang J, Ruddy DA, Sym M, et al (2002) aph-1 and pen-2 are required for Notch pathway signaling, gamma-secretase cleavage of betaAPP, and presenilin protein accumulation. Dev Cell 3: 85–97. 12110170 10.1016/s1534-5807(02)00189-2

[64] Goutte C, Tsunozaki M, Hale VA, Priess JR (2002) APH-1 is a multipass membrane protein essential for the Notch signaling pathway in Caenorhabditis elegans embryos. Proc Natl Acad Sci USA 99: 775–779. 10.1073/pnas.022523499 11792846 PMC117381

[65] Morel V, Schweisguth F (2000) Repression by suppressor of hairless and activation by Notch are required to define a single row of single-minded expressing cells in the Drosophila embryo. Genes Dev 14: 377–388. 10673509 PMC316365

[66] Hsieh JJ, Henkel T, Salmon P, Robey E, Peterson MG, et al (1996) Truncated mammalian Notch1 activates CBF1/RBPJk-repressed genes by a mechanism resembling that of Epstein-Barr virus EBNA2. Mol Cell Biol 16: 952–959. 8622698 10.1128/mcb.16.3.952 PMC231077

[67] Wesley CS, Mok L-P (2003) Regulation of Notch signaling by a novel mechanism involving suppressor of hairless stability and carboxyl terminus-truncated notch. Mol Cell Biol 23: 5581–5593. 12897132 10.1128/MCB.23.16.5581-5593.2003 PMC166347

[68] Kidd S, Lieber T, Young MW (1998) Ligand-induced cleavage and regulation of nuclear entry of Notch in Drosophila melanogaster embryos. Genes Dev 12: 3728–3740. 9851979 10.1101/gad.12.23.3728 PMC317253

[69] Wu L, Aster JC, Blacklow SC, Lake R, Artavanis-Tsakonas S, et al (2000) MAML1, a human homologue of Drosophila mastermind, is a transcriptional co-activator for NOTCH receptors. Nat Genet 26: 484–489. 10.1038/82644 11101851

[70] Kankel MW, Hurlbut GD, Upadhyay G, Yajnik V, Yedvobnick B, et al (2007) Investigating the genetic circuitry of mastermind in Drosophila, a notch signal effector. Genetics 177: 2493–2505. 10.1534/genetics.107.080994 18073442 PMC2219471

[71] Vied C, Kalderon D (2009) Hedgehog-stimulated stem cells depend on non-canonical activity of the Notch co-activator Mastermind. Development 136: 2177–2186. 10.1242/dev.035329 19474148 PMC2729338

[72] McElhinny AS, Li J-L, Wu L (2008) Mastermind-like transcriptional co-activators: emerging roles in regulating cross talk among multiple signaling pathways. Oncogene 27: 5138–5147. 10.1038/onc.2008.228 18758483

[73] Sestan N, Artavanis-Tsakonas S, Rakic P (1999) Contact-dependent inhibition of cortical neurite growth mediated by notch signaling. Science 286: 741–746. 10531053 10.1126/science.286.5440.741

[74] Franklin JL, Berechid BE, Cutting FB, Presente A, Chambers CB, et al (1999) Autonomous and non-autonomous regulation of mammalian neurite development by Notch1 and Delta1. Curr Biol 9: 1448–1457. 10607588 10.1016/s0960-9822(00)80114-1

[75] Berezovska O, McLean P, Knowles R, Frosh M, Lu FM, et al (1999) Notch1 inhibits neurite outgrowth in postmitotic primary neurons. Neuroscience 93: 433–439. 10465425 10.1016/s0306-4522(99)00157-8

[76] Levy OA, Lah JJ, Levey AI (2002) Notch signaling inhibits PC12 cell neurite outgrowth via RBP-J-dependent and-independent mechanisms. Dev Neurosci 24: 79–88. 12145413 10.1159/000064948

[77] Hassan BA, Bermingham NA, He Y, Sun Y, Jan YN, et al (2000) atonal regulates neurite arborization but does not act as a proneural gene in the Drosophila brain. Neuron 25: 549–561. 10774724 10.1016/s0896-6273(00)81059-4

[78] Knaden M, Strutz A, Ahsan J, Sachse S, Hansson BS (2012) Spatial representation of odorant valence in an insect brain. Cell Reports 1: 392–399. 10.1016/j.celrep.2012.03.002 22832228

[79] Schlief ML, Wilson RI (2007) Olfactory processing and behavior downstream from highly selective receptor neurons. Nat Neurosci 10: 623–630. 10.1038/nn1881 17417635 PMC2838507

[80] Carlsson MA, Diesner M, Schachtner J, Nässel DR (2010) Multiple neuropeptides in the Drosophila antennal lobe suggest complex modulatory circuits. J Comp Neurol 518: 3359–3380. 10.1002/cne.22405 20575072

[81] Yew JY, Wang Y, Barteneva N, Dikler S, Kutz-Naber KK, et al (2009) Analysis of neuropeptide expression and localization in adult drosophila melanogaster central nervous system by affinity cell-capture mass spectrometry. J Proteome Res 8: 1271–1284. 10.1021/pr800601x 19199706 PMC2693453

[82] Nässel DR, Winther AME (2010) Drosophila neuropeptides in regulation of physiology and behavior. Prog Neurobiol 92: 42–104. 10.1016/j.pneurobio.2010.04.010 20447440

[83] Heitzler P (2010) Biodiversity and noncanonical Notch signaling. Curr Top Dev Biol 92: 457–481. 10.1016/S0070-2153(10)92014-0 20816404

[84] Cingolani LA, Goda Y (2008) Actin in action: the interplay between the actin cytoskeleton and synaptic efficacy. Nat Rev Neurosci 9: 344–356. 10.1038/nrn2373 18425089

[85] Dillon C, Goda Y (2005) The actin cytoskeleton: integrating form and function at the synapse. Annu Rev Neurosci 28: 25–55. 10.1146/annurev.neuro.28.061604.135757 16029114

[86] Lamprecht R, LeDoux J (2004) Structural plasticity and memory. Nat Rev Neurosci 5: 45–54. 10.1038/nrn1301 14708003

[87] Giniger E (1998) A role for Abl in Notch signaling. Neuron 20: 667–681. 9581760 10.1016/s0896-6273(00)81007-7

[88] Crowner D, Le Gall M, Gates MA, Giniger E (2003) Notch steers Drosophila ISNb motor axons by regulating the Abl signaling pathway. Curr Biol 13: 967–972. 12781136 10.1016/s0960-9822(03)00325-7

[89] Sibbe M, Förster E, Basak O, Taylor V, Frotscher M (2009) Reelin and Notch1 cooperate in the development of the dentate gyrus. J Neurosci 29: 8578–8585. 10.1523/JNEUROSCI.0958-09.2009 19571148 PMC6665659

[90] Hashimoto-Torii K, Torii M, Sarkisian MR, Bartley CM, Shen J, et al (2008) Interaction between Reelin and Notch signaling regulates neuronal migration in the cerebral cortex. Neuron 60: 273–284. 10.1016/j.neuron.2008.09.026 18957219 PMC2913541

[91] Huang W, Zhu PJ, Zhang S, Zhou H, Stoica L, et al (2013) mTORC2 controls actin polymerization required for consolidation of long-term memory. Nat Neurosci 16: 441–448. 10.1038/nn.3351 23455608 PMC3615448

[92] Tan Y, Yu D, Busto GU, Wilson C, Davis RL (2013) Wnt signaling is required for long-term memory formation. Cell Reports 4: 1082–1089. 10.1016/j.celrep.2013.08.007 PMC4083693 24035392

[93] Twick I, Lee JA, Ramaswami M (2014) Olfactory habituation in Drosophila-odor encoding and its plasticity in the antennal lobe. Prog Brain Res 208: 3–38. 10.1016/B978-0-444-63350-7.00001–2 24767477

[94] Ni J-Q, Zhou R, Czech B, Liu L-P, Holderbaum L, et al (2011) A genome-scale shRNA resource for transgenic RNAi in Drosophila. Nat Meth 8: 405–407. 10.1038/nmeth.1592 PMC3489273 21460824

[95] Pfeiffer BD, Ngo T-TB, Hibbard KL, Murphy C, Jenett A, et al (2010) Refinement of tools for targeted gene expression in Drosophila. Genetics 186: 735–755. 10.1534/genetics.110.119917 20697123 PMC2942869

[96] Nagel AC, Maier D, Preiss A (2002) Green fluorescent protein as a convenient and versatile marker for studies on functional genomics in Drosophila. Dev Genes Evol 212: 93–98. 10.1007/s00427-002-0210-y 11914941

[97] Lee T, Luo L (1999) Mosaic analysis with a repressible cell marker for studies of gene function in neuronal morphogenesis. Neuron 22: 451–461. 10197526 10.1016/s0896-6273(00)80701-1

[98] Presente A, Shaw S, Nye JS, Andres AJ (2002) Transgene-mediated RNA interference defines a novel role for notch in chemosensory startle behavior. Genesis 34: 165–169. 10.1002/gene.10149 12324975

[99] Kennerdell JR, Carthew RW (2000) Heritable gene silencing in Drosophila using double-stranded RNA. Nat Biotechnol 18: 896–898. 10.1038/78531 10932163

[100] Pfeiffer BD, Truman JW, Rubin GM (2012) Using translational enhancers to increase transgene expression in Drosophila. Proc Natl Acad Sci USA 109: 6626–6631. 10.1073/pnas.1204520109 22493255 PMC3340069

[101] Tanaka NK, Awasaki T, Shimada T, Ito K (2004) Integration of chemosensory pathways in the Drosophila second-order olfactory centers. Curr Biol 14: 449–457. 10.1016/j.cub.2004.03.006 15043809

[102] Xiong WC, Okano H, Patel NH, Blendy JA, Montell C (1994) repo encodes a glial-specific homeo domain protein required in the Drosophila nervous system. Genes Dev 8: 981–994. 7926782 10.1101/gad.8.8.981

[103] Das A, Sen S, Lichtneckert R, Okada R, Ito K, et al (2008) Drosophila olfactory local interneurons and projection neurons derive from a common neuroblast lineage specified by the empty spiracles gene. Neural Dev 3: 33 10.1186/1749-8104-3-33 19055770 PMC2647541

[104] Dietzl G, Chen D, Schnorrer F, Su K-C, Barinova Y, et al (2007) A genome-wide transgenic RNAi library for conditional gene inactivation in Drosophila. Nature 448: 151–156. 10.1038/nature05954 17625558

[105] Merdes G, Soba P, Loewer A, Bilic MV, Beyreuther K, et al (2004) Interference of human and Drosophila APP and APP-like proteins with PNS development in Drosophila. EMBO J 23: 4082–4095. 10.1038/sj.emboj.7600413 15385958 PMC524346

[106] Datta SR, Vasconcelos ML, Ruta V, Luo S, Wong A, et al (2008) The Drosophila pheromone cVA activates a sexually dimorphic neural circuit. Nature 452: 473–477. 10.1038/nature06808 18305480

[107] Wang JW, Wong AM, Flores J, Vosshall LB, Axel R (2003) Two-photon calcium imaging reveals an odor-evoked map of activity in the fly brain. Cell 112: 271–282. 12553914 10.1016/s0092-8674(03)00004-7

[108] Caron SJC, Ruta V, Abbott LF, Axel R (2013) Random convergence of olfactory inputs in the Drosophila mushroom body. Nature 497: 113–117. 10.1038/nature12063 23615618 PMC4148081

[109] Wagh DA, Rasse TM, Asan E, Hofbauer A, Schwenkert I, et al (2006) Bruchpilot, a protein with homology to ELKS/CAST, is required for structural integrity and function of synaptic active zones in Drosophila. Neuron 49: 833–844. 10.1016/j.neuron.2006.02.008 16543132

