# Supplementary figures and images for: Notch Is Required in Adult Drosophila Sensory Neurons for Morphological and Functional Plasticity of the Olfactory Circuit
Source: PLoS Genet. 2015 May 26;11(5):e1005244. doi: 10.1371/journal.pgen.1005244 (PMC4444342; doi:10.1371/journal.pgen.1005244)

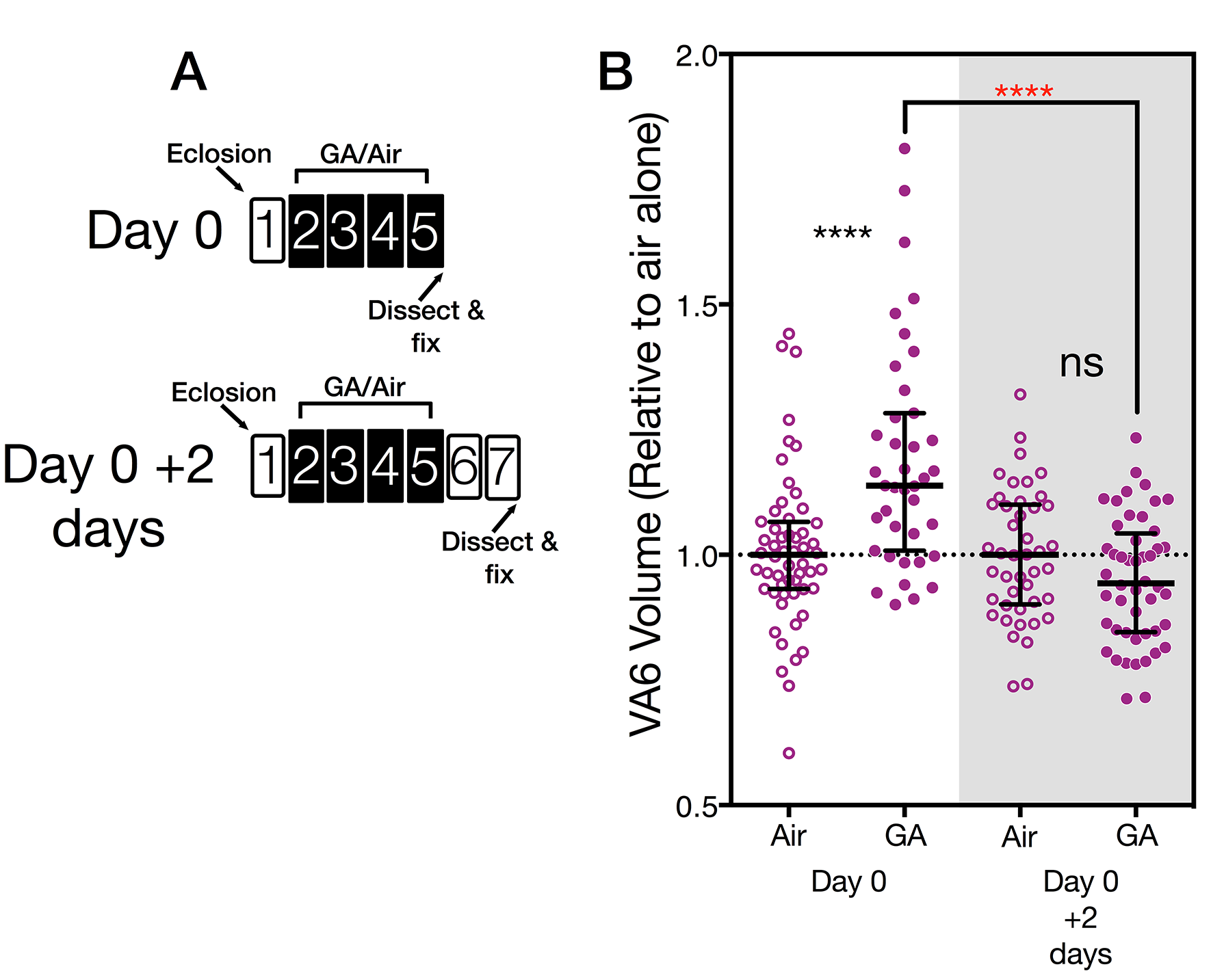

Supplement: S1 Fig — (A) Schematic of experimental protocol. One day old females expressing CD8-GFP and mCherry shRNA in VA6 ORNs under control of Or82a-GAL4 were exposed to a 1:100 dilution of GA or oil for 4 days. Flies were dissected immediately upon removal from odor (Day 0) or removed from odor and exposed to oil for 2 days prior to dissection (Day 0 + 2 days). (B) Scatter plots showing the volumes of VA6 glomeruli from air exposed (open circles) or GA exposed (filled circles) flies. Each circle represents the volume of a single glomerulus. The scatter plots are presented with median and interquartile ranges and were compared by Mann-Whitney tests. ns p>0.05; **** p≤0.0001. The volumes of all the glomeruli from each air/GA exposed pair were normalized to the median volume of the glomeruli of air exposed flies. The median volume of the glomeruli of the air exposed flies is by definition 1 and is indicated by the dashed line. This normalization allows us to compare the volumes of the GA exposed flies. The uppermost, red, p-value refers this comparison. The black p-value compares air exposed flies with the corresponding GA exposed flies and is shown directly above each pair. (TIFF) [file pgen.1005244.s001.tiff]

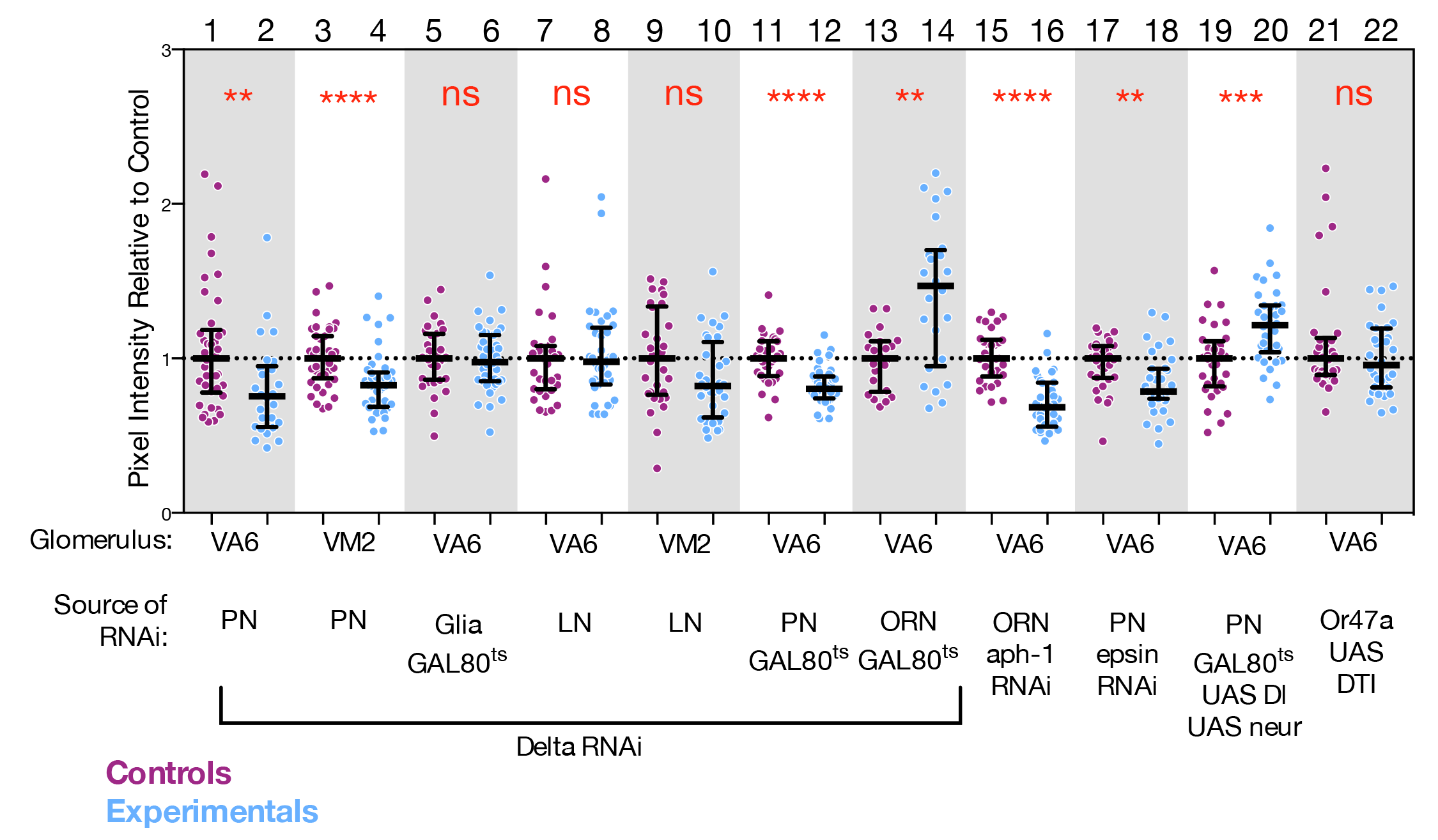

Supplement: S2 Fig — Scatter plots showing N reporter activity in the indicated glomeruli of odor exposed control flies (odd numbered lanes, purple) or flies expressing the indicated transgenes (even numbered lanes, blue) in either ORNs or PNs. Each circle represents the activity of a single glomerulus. The scatter plots are presented with median and interquartile ranges and were compared by Mann-Whitney tests. ns p>0.05; ** p≤0.01; *** p≤0.001; **** p≤0.0001. Each set of two lanes represents control flies and mutant flies from one experiment. For each GAL4 driver, reporter activity of all the glomeruli was normalized to the median reporter activity of the control flies. The median reporter activity of the control flies is by definition 1 and is indicated by the dashed line. The p-value for this comparison is indicated in red above each pair. N-LV LexOP.dGFP females carrying either UAS.Dl shRNA (lanes 2, 4, 6, 8 & 10), UAS.DlXK8 long inverted repeat RNA (lanes 12 & 14), UAS.aph-1 shRNA (lane 16), UAS.epsin RNAi (lane 18), UAS.Dl and UAS.neur (lane 20), or UAS.DTI [56] (lane 22) in the indicated cell type were exposed to a 1:100 dilution of GA (VA6), EB (VM2) or oil for 4 days. In lanes 5, 6, 11, 12, 13, 14, 19 and 20 the flies also carried tubP-gal80ts to limit expression to adults, and in lanes 11–14, 17 and 18 the flies also carried UAS.dicer-2 (dcr) to potentiate the RNAi. The control flies in lanes 1, 3, 5, 7, 9 & 15 expressed mCherry shRNA which is inserted into the same chromosomal location as the indicated transgenes. In lanes 11, 13, 17, 19 & 21 the controls lacked the indicated RNAi (lanes 11, 13 & 17), or the indicated UAS constructs (lanes 19 & 21). The following GAL4 drivers were used: VA6 PNs, MZ612; VM2 PNs, NP5103; VA6 glia, repo; VA6 and VM2 LNs, NP2426; VA6 ORNs, Or82a-GAL4. (TIF) [file pgen.1005244.s002.tif]

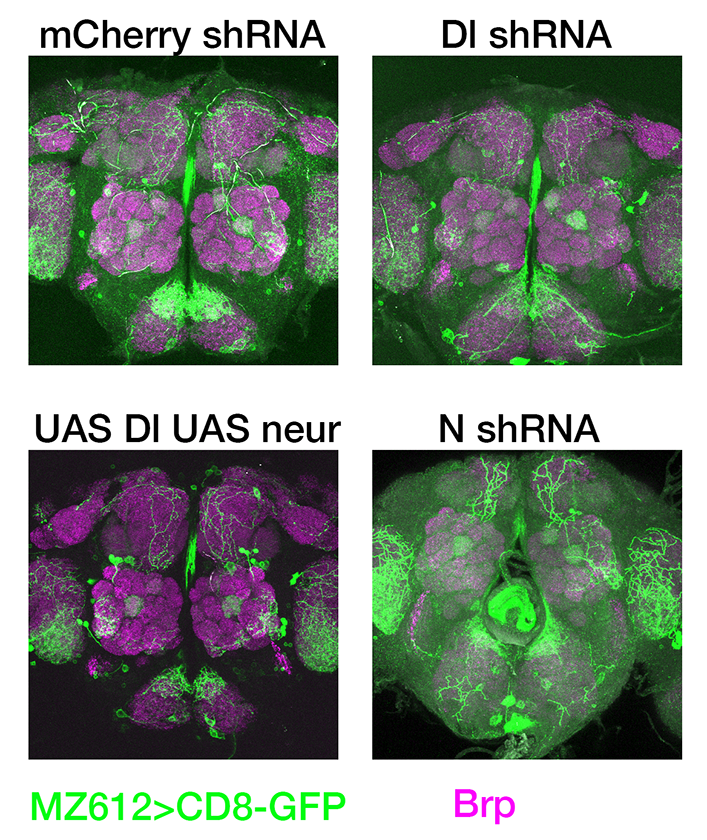

Supplement: S3 Fig — Brains of flies expressing CD8-GFP and the indicated transgenes in VA6 PNs, under control of MZ612-GAL4, were stained with anti-GFP (green) and anti-Bruchpilot (Brp (nc82), magenta) antibodies. In all cases, CD8-GFP expression can be detected in VA6 indicating that expression of the transgenes does not affect projection of VA6 PNs to VA6. MZ612-GAL4 is not expressed exclusively in VA6 PNs. (TIF) [file pgen.1005244.s003.tif]

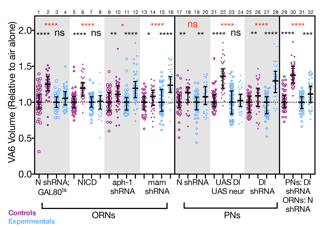

Supplement: S4 Fig — Scatter plots showing the volumes of VA6 glomeruli in control flies (purple) or flies expressing the indicated transgenes (blue) in either ORNs or PNs. Each symbol represents the volume of a single glomerulus. The scatter plots are presented with median and interquartile ranges and were compared by Mann-Whitney tests. ns p>0.05; * p≤0.05; ** p≤0.01; *** p≤0.001; **** p≤0.0001. Each set of four lanes represents control flies (purple) and mutant flies (blue) from one experiment. The flies in the odd numbered lanes (open symbols) were exposed to air, and the flies in the even numbered lanes (filled symbols) were chronically exposed to GA. The volumes of all the glomeruli from each air/GA exposed pair were normalized to the median volume of the glomeruli of air exposed flies. The median volume of the glomeruli of the air exposed flies is by definition 1 and is indicated by the dashed line. This normalization allows us to compare the volumes of the GA exposed flies in each experiment. The uppermost, red, p-value refers this comparison. The black p-value compares air exposed flies with the corresponding GA exposed flies of the same genotype and is shown directly above each pair. The indicated transgenes were expressed in either VA6 ORNs under control of Or82a-GAL4 (lanes 1–16) or VA6 PNs under control of MZ612-GAL4 (lanes 17–28). Flies in lanes 1–16 carry UAS.CD8-GFP. Flies in lanes 17–28 carry Or82a.CD8-GFP. Flies in lanes 1–4 carry tubP-gal80ts10. Flies in lanes 29–32 express Dl RNAi in VA6 PNs under control of MZ612-GAL4, and LexAop.cd8-GFP together with either control (lane 29 and 30) or NRNAi (lane 31 and 32) in VA6 ORNs under control of Or82a-LexAGAD. The controls are mCherry shRNA (lanes 1, 2, 5, 6, 9, 10, 13, 14, 17, 18, 25, 26, 29 and 30), which is inserted into the same chromosomal location as the indicated transgenes. In lane 21 & 22 the flies lacked the indicated UAS constructs. Lanes 1–4 depict the same data as in Fig 2 lanes 1–4. (TIFF) [file pgen.1005244.s004.tiff]

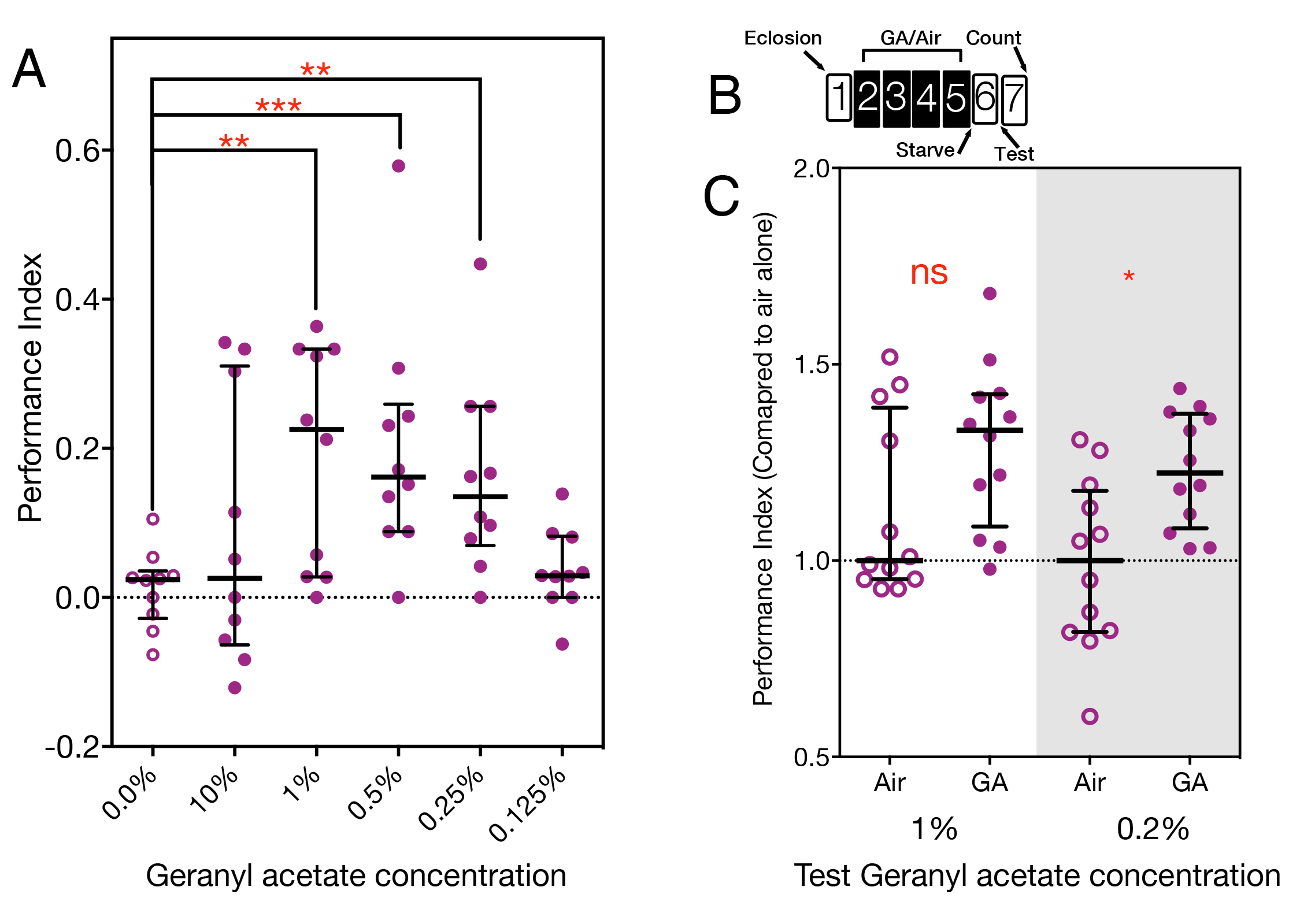

Supplement: S5 Fig — (A) Flies are attracted to GA. Five day old female flies expressing mCherry RNAi in VA6 ORNs under control of Or82a-GAL4 were starved for 24 hours and then placed in mesh covered beakers in the dark, where they had a choice between scintillation vials with the indicated concentrations of GA in 0.1% triton or solvent alone. The flies were counted at 24 hrs. Performance Index (PI) is (GA—solvent)/(total flies). Each point represents the PI of a single beaker, with 40 flies per beaker. Here and in C the scatter plots are presented with median and interquartile ranges and were compared by Mann-Whitney tests. ** p≤0.01; *** p≤0.001; non-significant differences are not marked. Or82a-GAL4 flies were outcrossed to cn bw flies for 6 generations. (B & C) Prolonged exposure to GA enhances attraction to subsequent presentation of GA. One day old female flies expressing mCherry RNAi in VA6 ORNs under control of Or82a-GAL4 were exposed on food to 1% GA in paraffin oil (GA, closed circles) or to paraffin oil alone (air, open circles) for 4 days. The flies were removed from odor, starved for 24 hours, and then placed in mesh covered beakers in the dark where they had a choice between 2% ethyl butyrate or the indicated concentrations of GA, all diluted in 0.1% triton. The flies were counted at 24 hours. PI is (GA—EB)/(total flies). Each point represents the PI of a single beaker, with 40 flies per beaker. In the log plots the larger the number the greater the attraction to GA. The data were normalized by dividing by the median of the air exposed flies for each concentration of GA. ns, p>0.05; *, p≤0.05. (TIF) [file pgen.1005244.s005.tif]

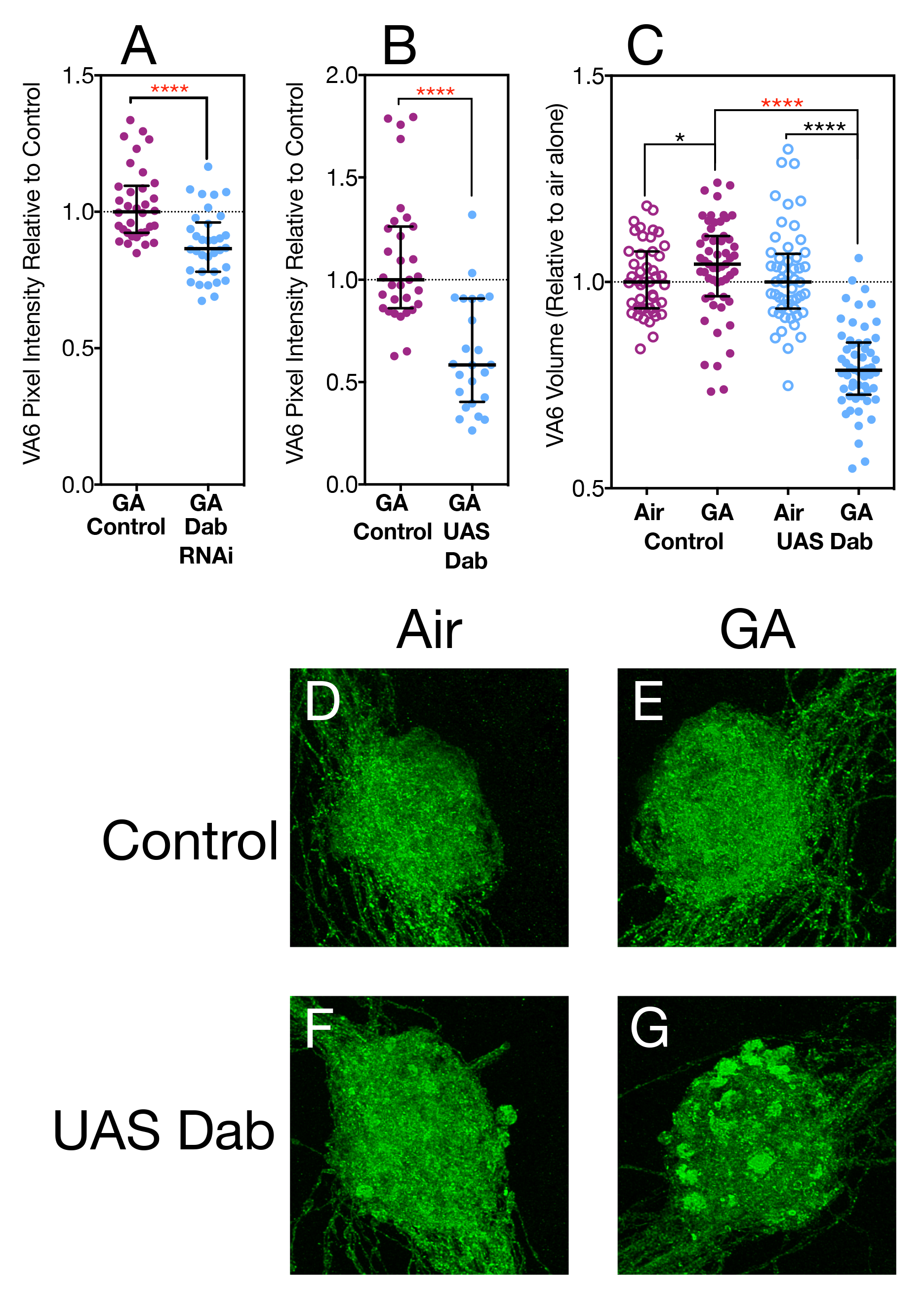

Supplement: S6 Fig — One day old female flies were exposed to 1% GA in paraffin oil (GA) or paraffin oil alone (air) for 4 days, after which (A & B) N reporter activity or (C) VA6 size was determined. In A & B the level of N reporter activity seen in odor exposed flies carrying the indicated transgenes was normalized to the N reporter activity seen in odor exposed controls. In C the volumes of all the glomeruli from each air/GA exposed pair were normalized to the median volume of the glomeruli of air exposed flies. This normalization allows us to compare the volumes of the GA exposed flies in each experiment. The uppermost, red, p-value refers this comparison. The black p-value compares air exposed flies with the corresponding GA exposed flies of the same genotype and is shown directly above each pair. * p≤0.05; *** p≤0.001; **** p≤0.0001. Panels D-G illustrate the changes in morphology and size caused by over-expression of Dab in conjunction with odor. (A) Or82a-GAL4; NLV LexOP.dGFP with mCherry (control) or Dab shRNA. (B) Or82a-GAL4; NLV LexOP.dGFP without (control) or with UAS.Dab. (C-G) Or82a-GAL4; UAS cd8-GFP without (control) or with UAS.Dab. (TIF) [file pgen.1005244.s006.tif]
